# Interrogating the promise of technology in epilepsy care: systematic, hermeneutic review

**DOI:** 10.1111/1467-9566.13266

**Published:** 2021-04-01

**Authors:** Chrysanthi Papoutsi, Christian D.E. Collins, Alexandra Christopher, Sara E. Shaw, Trisha Greenhalgh

**Affiliations:** ^1^ Nuffield Department of Primary Care Health Sciences University of Oxford Oxford UK; ^2^ North Oxfordshire GP Training Scheme, Terence Mortimer Education Centre Horton General Hospital Banbury Oxfordshire UK

**Keywords:** epilepsy, e‐health, hermeneutic review, sociotechnical theory, user representations

## Abstract

Technology development is gathering pace in epilepsy with seizure detection devices promising to transform self‐care and service provision. However, such accounts often neglect the uncertainties, displacements and responsibilities that technology‐supported care generates. This review brings together a heterogeneous literature, identified through systematic searches in 8 databases and snowball searching, to interrogate how technology becomes positioned in epilepsy care. We took a hermeneutic approach in our analysis of the 206 included articles, which resulted in the development of a conceptual framework surfacing the underlying logics by which technology‐supported epilepsy care is organised. Each of these logics enacts different techno‐scientific futures and carries specific assumptions about how (often imagined) ‘users’ and their bodies become co‐constituted. Our review shows that studies in this area remain primarily deterministic and technology‐focused. Few draw phenomenological insights on lived experiences with epilepsy or use social theory to problematise the role of technology. We propose future directions for sociotechnical, theory‐driven studies of technology in epilepsy care and offer a framework transferable across other long‐term conditions.

## INTRODUCTION

Epilepsy was once the poster child of medical sociology. In the 1980s, it was the focus of seminal theoretical publications on stigma (Scambler, 1989), and sociological versus biomedical models of illness (Schneider & Conrad, 1981). Epilepsy‐related research has continued to the present day (Shostak & Fox, 2012; Walker et al., 2014; Webster, 2019; Weckesser et al., 2017), but the field has yet to embrace an important development in contemporary epilepsy care – the use of technology to monitor and manage the condition. This contrasts with recent theorisations of technology in (for example) heart failure (Pols, 2012), ageing (Brittain et al., 2010) and healthy individuals (Lupton, 2013).

Epilepsy affects 70 million people worldwide and accounts for 14.8 million disability‐adjusted life‐years and 130,000 deaths per year (Thijs et al., 2019). This neurological condition comprises a varied manifestation of multiple pathologies leading to different kinds of seizures (Rai et al., 2012; Thijs et al., 2019). With careful monitoring and personalised adjustment of anti‐epileptic treatments, two‐thirds of patients in high‐income settings have the potential to become well‐controlled and largely seizure‐free, although still experiencing significant side effects, including drug toxicity (Macfarlane & Greenhalgh, 2018; Thijs et al., 2019). Epilepsy has a profound impact on people’s lives: constant fear of seizure onset often leads to social isolation (Jacoby et al., 2005; Ryan & Raisanen, 2012; Stepney et al., 2018); patients report cognitive difficulties, emotional distress, frustration, low mood and anxiety (Kerr et al., 2011); they face underemployment and unemployment (Smeets et al., 2007); and experience significant stigma (Jacoby et al., 2005; Scambler, 1989).

Different technologies are available in epilepsy care, including wearables for seizure monitoring, seizure diary apps, online patient education programmes and implantable treatment devices (Horizon Scanning Research & Intelligence Centre, 2017). Some of these technologies are already well established; others are currently under development by public and private organisations across the world (Escoffery et al., 2018; Van de Vel et al., 2016). Yet, there has been little sociological research on the role new technologies play in epilepsy care. This hermeneutic review follows the trail of patient‐facing technologies in the published literature, to make visible the multiple ways in which technology is being framed, and articulate what this means for the mutual shaping of devices, users and bodies living with epilepsy (Berg & Harterink, 2004; Mort et al., 2009).

In doing this, we engage with the enduring appeal of technological determinism (and its different articulations), to understand why it ‘remains in the justification of actors who are keen to promote a particular direction of change’ (Wyatt, 2008, p.176). Our starting point is in unpacking technological determinism as an ‘ideology’ (Edwards, 1995), which continues to be strongly embedded in depictions of technology in health care. ‘Modernist’ discourses about health technologies have been criticised for their instrumentality and for equating technological development with progress (Greenhalgh et al., 2012). More nuanced views have instead emphasised technology as (often ambivalently) embedded in social, political and economic relations (e.g. Timmermans & Berg, 2003; Woolgar & Cooper, 1999; Wyatt, 2008).

Webster has long talked about how the promise of health technologies relies partly on relegating uncertainty and ‘mobilising a range of claims about their future therapeutic impact’ (Webster, 2002, p.443). Representations of the future, filled with hope about the potential of technology, act performatively to enrol actors in the innovation network and to stabilise the support needed (Borup et al., 2006; Brown & Michael, 2003). It becomes important to unpack this anticipatory and promissory work, to understand what representations may be missing, whether specific worldviews succeed in co‐shaping the development process, and how a better balance could be achieved.

### Technology perspectives and user representations

Various theorisations of the user‐technology nexus have emerged in social studies of technology development (Oudshoorn & Pinch, 2003; Suchman, 2007). Relationships between users and machines have been described as ‘configured’ in that design parameters enable and contain only a certain range of user actions (Grint & Woolgar, 1997). Others have focused on the way explicit and implicit user representations become ‘inscribed’ in technologies during design (Akrich, 1992; Akrich, 1995). Although criticised as linear depictions of technology development, both perspectives recognise that ‘scripts’ will not necessarily be ‘read’ as intended by designers and that actual use will not always match design expectations (Oudshoorn & Pinch 2003). Yet, it is important to surface what de Laat calls fictive scripts as ‘the emergence of a new technology always depends on those developing it making assumptions about its future location in a wider technological ecology’ (De Laat, 2000, p.9).

More recent work extends beyond the notion of ‘envisioned’ users in design practices to emphasise the dynamics of co‐constitution between users and design practices (Hyysalo et al., 2016; Oudshoorn & Pinch, 2003; Stewart & Williams, 2005). It highlights the heterogeneous and competing representations in material technologies that often derive from pre‐existing repertoires and are influenced by multiple actors beyond technology developers (Hyysalo et al., 2016; Hyysalo & Johnson, 2016; Oudshoorn & Pinch, 2003). In a similar vein, Suchman (2007) looks at how ‘capacities for action’ are distributed across different human–machine configurations. The related notion of ‘usership’ points to the practices through which social actors become ‘constructed as’ and ‘make themselves into device users’, taking into account structural influences (e.g. political and industrial economy) but also the varied configurability of different technological practices (Faulkner, 2009, p.19).

Beyond user representations, a diverse literature has grappled with the way bodies become implicated with technologies (Haraway, 1991; Latimer & Schillmeier, 2009; Lupton, 2017; Webster, 2002). Specific accounts of the body are reproduced in the context of medical technologies that support collaboratively accomplished professional work (Berg & Bowker, 1997; Hartswood et al., 2002). Bodies are increasingly seen as embedded in assemblages with knowledge and affective practices (Fox, 2017; Lupton, 2015). Multiple body ontologies and other subjectivities become co‐constituted within technological practices (Berg & Harterink, 2004; Berg & Mol, 1998); for example, in terms of how different treatment practices produce different versions of the medical condition and different ontologies of bodily organs affected (Willems, 1998); or how bodies and diseases come into being, not as stable, independent entities, but within sociomaterial practices (Mol, 2002; Mol & Law, 2004).

Taking analytical cues from the theoretical literature summarised above, in this article we present a review of published articles on patient‐facing technologies in epilepsy and examine how these accounts position their subject matter. We did not view technologies, users and bodies as separate entities, but as part of a nexus, being interested in how patients are imagined as users of these technologies and how their bodies are implicated in this process (Berg & Harterink, 2004; Mort et al., 2009; Webster, 2002). Our goal was to ‘*maximise what we will see*’ in a theoretically informed way rather than provide yet another summary of the literature (Weick, 1987, p.122). The review was guided by the following questions: What types of patient‐facing technologies have been used or are being developed in epilepsy care? How have previous studies framed such technologies and their intended users? What are the strengths and limitations of these framings – and how might new or different framings help inform development of technology‐supported epilepsy care?

## METHODS

**FIGURE 1:**
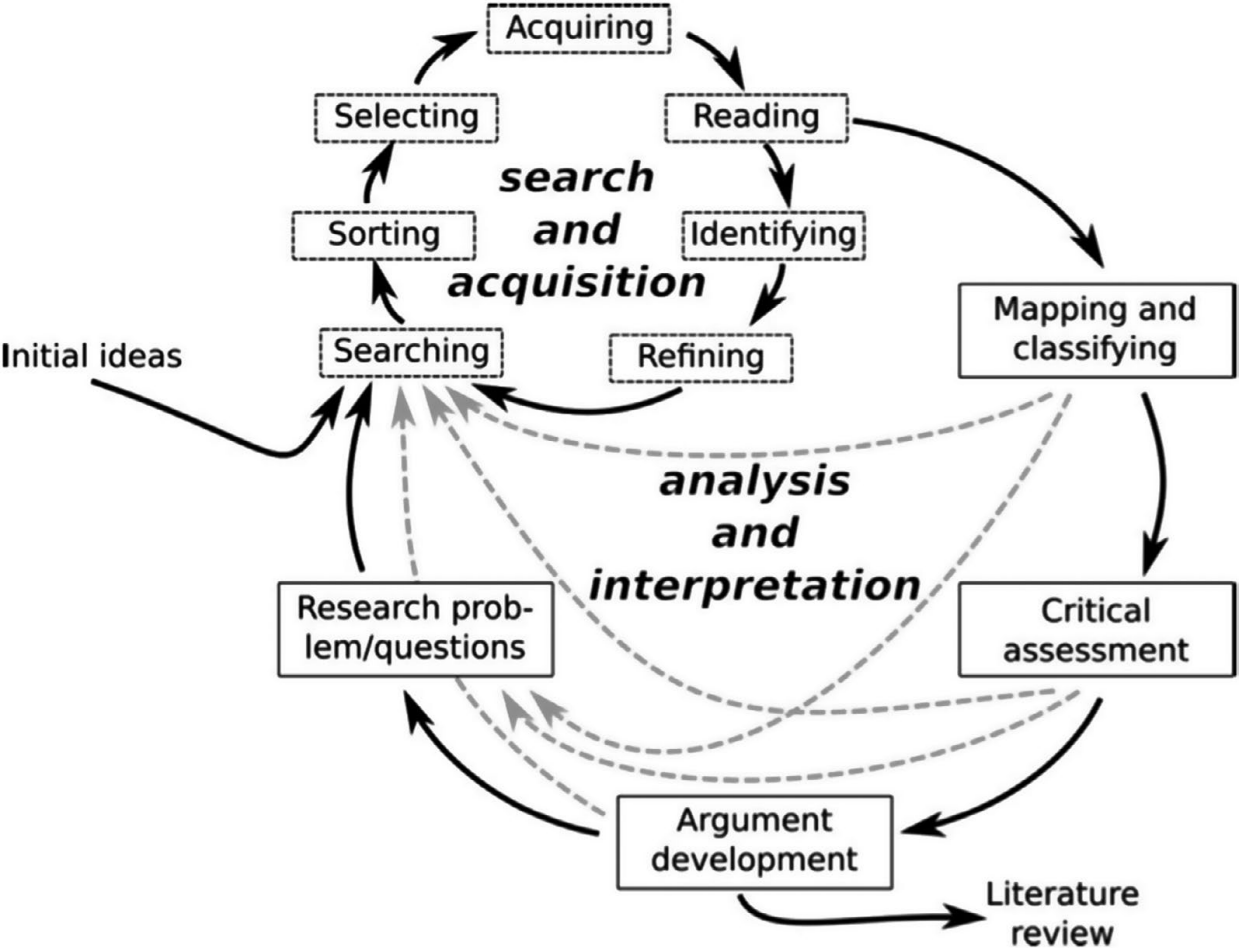
Iterative processes involved in a hermeneutic review. Reproduced with permission from Boell & Cecez‐Kecmanovic (2014)

We used a hermeneutic approach (illustrated in Figure 1) characterised by iterative exploration of, and critical engagement with, the literature (Boell & Cecez‐Kecmanovic, 2014).

### Search strategy and screening

With the help of an information specialist, we carried out a systematic search on epilepsy and technology. As this resulted in few examples of lived experiences using technologies, we performed a second systematic search looking for qualitative studies of patient experiences living with epilepsy (both searches were originally conducted in 2017 and updated in March 2019; see supplementary files). We searched 5 databases for both searches (Cinahl, Embase, Ovid MEDLINE (including ahead of print), PsycINFO, Science Citation Index, Social Science Citation Index & Conference Proceedings Citation Index – Science, Social Sciences & Humanities) and 3 additional databases for search 1 (Cochrane Database of Systematic reviews, Cochrane Central Register of Controlled Trials, Database of Abstracts of Reviews of Effects).

We iteratively sought additional articles by searching reference lists and tracking citations of seminal articles, alongside expert recommendations (Greenhalgh & Peacock, 2005). Using the Covidence platform (www.covidence.org), three reviewers screened titles and abstracts from search 1 (CC, CP, AC) and two reviewers screened articles from search 2 (CP, AC). We followed the same process to screen full texts of remaining articles. We collectively discussed agreements, disagreements and doubts to inform our hermeneutic understanding (using flexible inclusion/exclusion criteria provided in Box 1). This process clarified and sharpened our focus, as we attempted to balance institutionalised demands of ‘systematic’ and ‘rigorous’ literature searching and screening, with the openness, curiosity and quest for hidden meanings that characterise a hermeneutic analysis.

BOX 1 Broad inclusion criteria (combined for both searches): studies on patient‐facing technologies† in epilepsy care, patient experiences living with epilepsy and/or using technologies for their care, all study designs and English language only.

Broad exclusion criteria (combined for both searches): studies on technologies other than patient‐facing, for other user groups (e.g. online education for nurses) or other conditions, studies purely describing technical components without discussing applications in epilepsy‐related technologies or implementation, paediatric population, unless the technologies were also applicable to adults, abstracts in the technology search only (unless they contributed significant information).

† Patient‐facing technologies: digital and/or information technologies for adoption and use primarily by patients, relatives or carers (excluding hospital diagnostics and neuromodulation devices).

### Analysis and interpretation

Two reviewers (CP, CC) read and analysed the texts in our data set, using NVivo 11 for data management. Initially, we separated articles in high and low relevance categories to facilitate the analytical process. Articles from search 2 were primarily used to build an understanding of broader concerns as articulated in the experiences of people living with epilepsy. In our ‘conversations’ with the different texts, we stayed ‘attuned and engaged’ to understand not just what was being said but also what was being backgrounded: ‘insight comes from stepping back to see from a distance, from reading between the lines, from a sudden grasp of a new way of seeing’ (Smythe & Spence, 2012, p.23). We have successfully applied the same approach in a previous review of heart failure technologies (Greenhalgh et al., 2017). Our interpretations were also informed by empirical research carried out in parallel in a specialist epilepsy clinic, where a combination of technologies were piloted (wearables, smartphone apps and back‐end clinical systems) to support epilepsy care and monitoring (e.g. see Papoutsi et al., 2020).

### Framework development

As our hermeneutic review developed, we were struck by the contrasting ways in which digital technologies for epilepsy had been conceptualised in our data set. Some of our findings resonated with the five views on technology proposed by Orlikowski and Iacono (2001): ‘nominal’ (technology mentioned only in name, but not explored); ‘computational’ (technology studied for specific algorithms, programmes or models); ‘proxy’ (research on attitudes to technology); ‘tool’ (technology researched for its role in labour substitution, productivity improvement or information processing); and ‘ensemble’ (technology studied as part of a dynamic system). Our analysis led us to extend and re‐work this framework in the context of health technologies, using epilepsy as an exemplar and placing new emphasis on the mutual shaping of technologies, users and bodies.

## RESULTS

### Overview of data set

**FIGURE 2:**
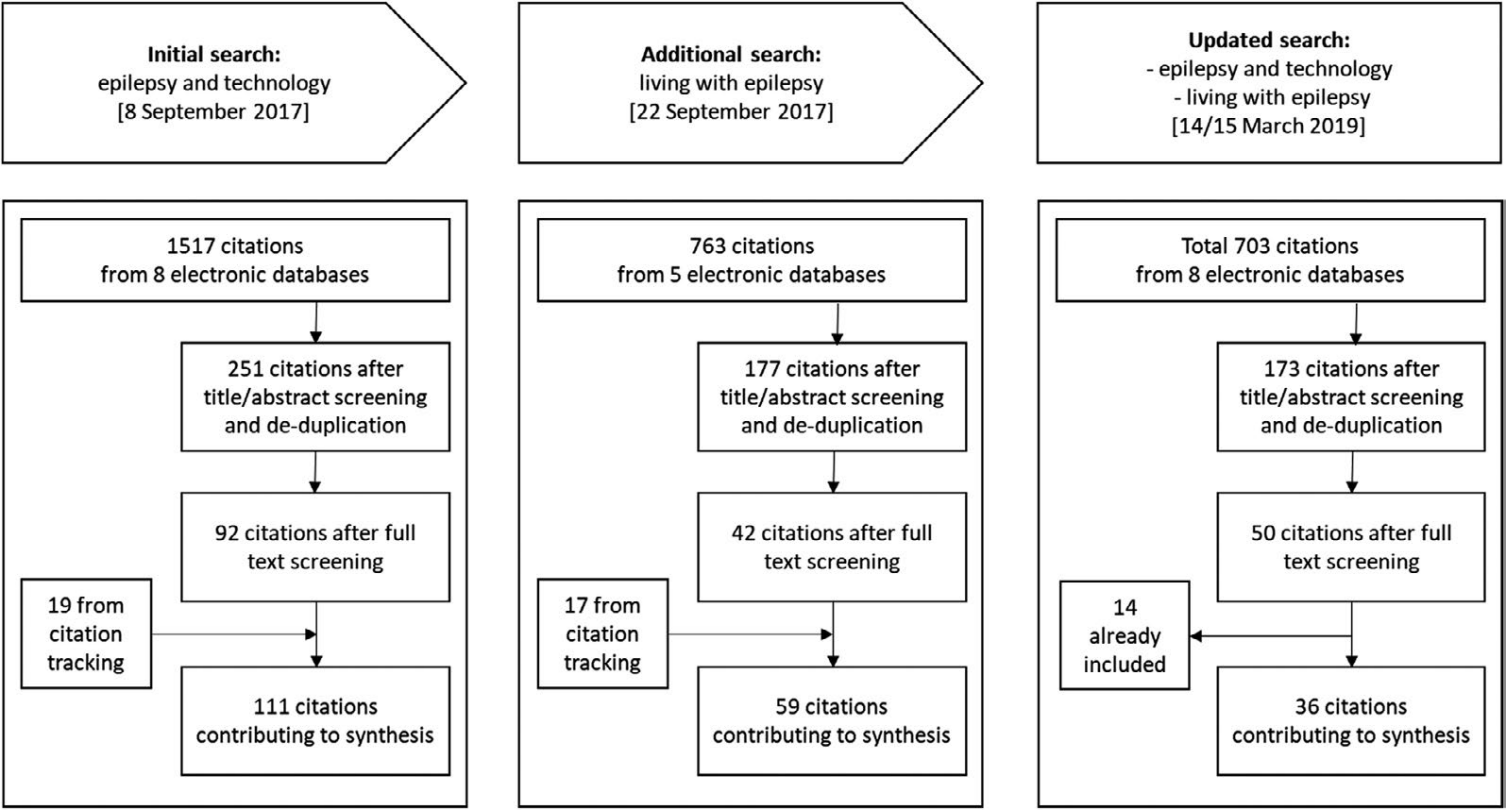
Study flowchart

The study flowchart, along with search and screening results, is shown in Figure 2. Of 2,983 titles, a total 206 articles were included in our data set: 134 from the original two searches in 2017, 36 from snowball searches and 36 from the updated search. Our data set was largely heterogeneous and included articles consisting of descriptions of technical architecture and algorithm development or testing (e.g. Geertsema et al., 2018^*^); efficacy studies or randomised controlled trials of technologies (Carlson et al., 2009^*^); feasibility and acceptability studies (e.g. Liu et al., 2016^*^); survey‐based research (Arthurs et al., 2010^*^); qualitative methods (Ozanne et al., 2017^*^); mixed methods (Leenen et al., 2017^*^); systematic, narrative or descriptive reviews (Jory et al., 2016^*^); and additional commentaries and editorials (Elger & Mormann, 2013^*^).

Studies of epilepsy technologies had been published almost exclusively in the clinical and technical literature and had not previously linked to the research literature on patient experiences living with epilepsy – an important omission, since good sociotechnical design relies on a detailed analysis of patient needs and contexts (Greenhalgh et al., 2015). The literature on epilepsy technologies focused on technical development in experimental settings, and there was minimal overlap (only 2 articles) in the results between search 1 (technologies for epilepsy) and search 2 (lived experiences of epilepsy). Qualitative studies on technology were reported primarily in the form of abstracts with limited detail.

Much hope was expressed in the potential of technology in epilepsy care, but there were tensions between this rhetoric and empirical findings, which generally identified limitations in the technologies tested. Most experimental studies were relatively small and short‐term (2–5 patients at pilot stage or 40–50 patients at more advanced stages), and survey studies were not always powered for advanced analysis (80–120 participants across most studies, with few exceptions). Research was carried out mainly in the USA (91 articles), in the UK (46 articles) and to a smaller extent in other countries (e.g. the Netherlands, Australia). Supplementary files provide the characteristics of all included articles.

Extending Orlikowski and Iacono (2001), we identified six different ways in which patient‐facing technologies for epilepsy had been presented in the literature:

1. COMPUTATIONAL VIEW: technology studied in terms of technical and computational properties.
2. TOOL VIEW: technology studied in terms of the extent to which it supports clinical tasks.
3. PLATFORM VIEW: technology studied primarily in terms of its ability to support self‐management, including by hosting exchange and interaction among patients.
4. INTERMEDIARY VIEW: technology studied in terms of mobilising data to make patients ‘knowable’ in certain ways, or supporting certain modes of patient care.
5. ATTITUDINAL VIEW: technology is not studied directly, but patients and other user groups are surveyed about their attitudes to technology.
6. DYNAMIC VIEW: technology studied within the context of a sociotechnical system, or as an actor embedded in a wider network.

Although distinct in their assumptions and implications, these logics are intended to illustrate multiplicity, in that a single article or technology often makes visible more than one ways of thinking. Beyond technology conceptualisations, these six lenses also surface different ways of imagining the role of patients as technology users and reveal different assumptions about how patients’ bodies are (implicitly or explicitly) accounted for in this literature. Table 1 elaborates on the above categories.

**TABLE 1:** Overview of approaches to technology in the literature on epilepsy care (2001–2019), commonly used research questions, and implications for the ways people living with epilepsy (as users and bodies) become positioned in these perspectives.

	Technology as ‘computational artefact’	Technology as ‘tool’	Technology as ‘platform’	Technology as ‘intermediary’	Attitudes to technology	Technology as part of a dynamic sociotechnical system
Technology viewed as	Having the potential to be optimised through programming for maximum effect, especially in terms of computational power and capability.	Directly and ‘objectively’ capturing data on the condition, enhances monitoring accuracy and allows better data aggregation and information processing based on pre‐defined measures.	As conduit for education, peer support, self‐monitoring and self‐management.	Intermediary providing easier access to patients, making them knowable by mobilising specific types of data and ways of connecting, and often transcending physical distance between clinical teams, patients, researchers.	Artefact (or imagined object) towards which potential users have more or less fixed attitudes (which are assumed to lead to either acceptance or rejection of the technology).	Actor or component in a wider network; a product of its conditions of development; enmeshed with the conditions of its use; implicated in sociocultural dynamics; embodying and recursively shaping social structures.
Typical format of research question	How can we technically optimise computational systems to represent, simulate and predict human activity?	What is the impact of the technology on patient monitoring, outcomes or clinical processes?	What is the efficacy and effectiveness of technology in improving self‐management?	How can technology be used to mediate interactions with patients?	Is the technology acceptable to users? What levels of satisfaction, patterns of use and diffusion exist? What individual factors account for non‐adoption or resistance to technology?	What complex relationships between actors sustain technology‐supported care? How do social influences shape technology introduction? How do social structures become embedded in technology appropriation?
Users positioned as	Largely absent, mostly understood as subject to the technology or beneficiary of increased computational capacity and performance.	Less reliable in reporting and self‐monitoring than the technology (e.g. not able to remember seizures in epilepsy). Technology thus compensates for human limitations.	Playing an active role in self‐care and self‐monitoring with emphasis on knowledge requirements and motivational needs.	Providing data input and participating in technology‐enhanced ways of caring.	Potential adopter or recipient who makes utility‐based, binary calculations in deciding to embrace or resist the technology.	Active, creative agent, enabled and constrained by wider social structures and material conditions.
The body depicted as	Subject to technical description and the basis on which to model computational activity and classification.	A site for technological intervention and enhancement.	Object to be represented on a digital platform and self‐managed by patients themselves with the appropriate education.	Quantifiable and a site of observation, rendered into disembodied digital data with technology use.	A site of technology use or resistance.	Active agent both shaping and shaped by technologies in practice.

### Technology as computational artefact

Despite having excluded results with a core technical focus, several articles that met inclusion criteria still framed technology as a computational artefact. These emphasised computational and algorithmic dimensions of epilepsy technologies, for example system modelling and architecture, testing and refinement of algorithms for patient‐facing applications, and biosignal processing. Focused on technical capabilities and algorithmic performance improvement, their accounts made little reference to the complexity of real‐world implementation, implying that technology would be meaningfully used as soon as technical challenges had been addressed. In this literature, patients were invoked primarily as prospective beneficiaries of technical advances and were largely absent from the design and development processes, other than as ‘test subjects’ in a small number of articles.

**FIGURE 3:**
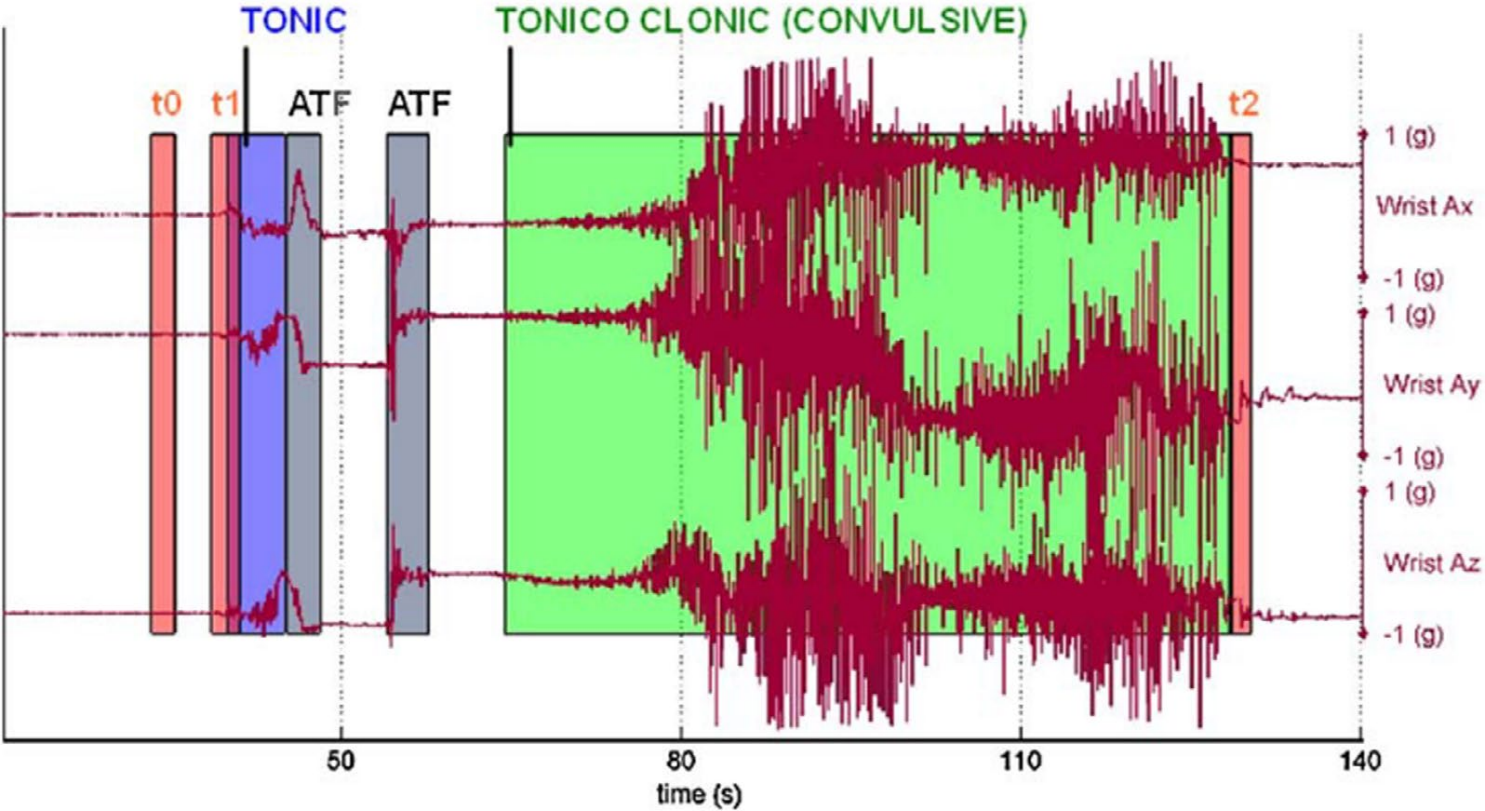
Representations of epileptic seizures in accelerometer graphs. Reproduced with permission from Bonnet et al. (2011^*^)

This computational and algorithmic lens foregrounded a view of patient bodies as sites of measurement and technical description. It typically isolated specific features of the condition, such as motor activity in certain types of seizures, and described them from a technical or algorithmic perspective. For example, Bonnet et al (2011^*^) captured bodily movements using body‐worn motion sensors which led to representations of epileptic seizures in accelerometer graphs (see Figure 3). Their article engaged with issues of ‘accuracy’ in measuring human body orientation and focused on the technical components that would allow this, for example ‘using miniaturized high‐tech motion sensing […] containing 3‐axis accelerometers (3a) and 3‐axis magnetometers (3m)’ (Bonnet et al., 2011, p.156^*^).

Several studies on seizure detection and monitoring systems examined whether the technology performed the technical tasks it was intended to do. They mapped out software, hardware characteristics and algorithmic implementation, including technical comparisons (Borujeny et al., 2013; Cogan et al., 2017; Dourado et al., 2009^*^). This literature primarily emphasised challenges of reducing computational load (Conradsen et al., 2012^*^), building ‘patient‐specific movement models’ from accelerometers rather than historic database data (Cuppens et al., 2014^*^) and developing models for the detection of seizures without motor activity (Heldberg et al., 2015^*^).

Behind such framing and language, the lived experience of seizures disappears to give precedence to the technical representation of bodily functions captured in highly specific ways. This could lead to a narrow focus on epilepsy monitoring purely as a technical problem, with wider challenges in technology use and adoption being neglected. Although testing is important to meet technical objectives, this needs to be combined with studies taking a broader view of technologies in complex personal and clinical contexts.

### Technology as tool

Articles in this subset employed a view of technology as a *tool* in the pursuit of various clinical (rather than technical) ends: monitoring seizures by translating bodily functions, sensations and experiences into mechanical signal; enhancing the effectiveness of care; and delivering treatment. Using feasibility and efficacy designs, they placed less focus on technical performance and architecture, and more attention on assessing potential contribution to clinical processes and outcomes. Their language and study designs, however, still reflected a deterministic view of how improvements would be achieved (as the quoted excerpts below suggest), by emphasising the promise of technology (e.g. new devices, sensor modalities or wireless transmission) over self‐care dynamics and the medical practices in which technology would be embedded.

**FIGURE 4:**
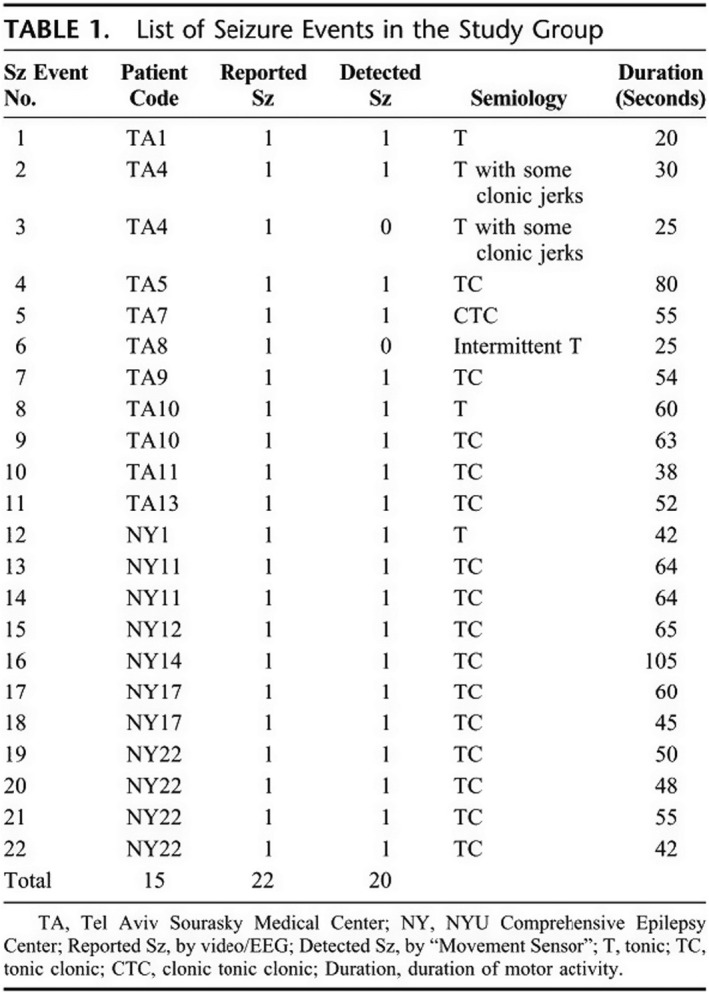
List of seizure events in the study group. Reproduced with permission from Kramer et al. (2011, p. 37^*^)

Sensitivity and specificity assessments dominated this literature with studies achieving variable levels of success (e.g. see Beniczky et al., 2013; Carlson et al., 2009; Kramer et al., 2011; Lockman et al., 2011; Patterson et al., 2015; Van de Vel et al., 2016b^*^). Instead of the accelerometer waveform in Figure 3, studies in this category typically portrayed seizures in terms of frequency and duration, with a view to inform clinical decision‐making by focusing on seizure management as a counting exercise (see Figure 4).

Although accurate detection of seizures has been achieved in laboratory, inpatient and other controlled settings with limited patient numbers, larger studies failed to replicate these results (Kramer et al., 2011; Lockman et al., 2011; Patterson et al., 2015^*^). Significant challenges have been identified outside controlled environments, primarily framed by this literature in technical terms, such as user ‘disruption’ from continuous sensing, high rates of false alarms, inaccurate real‐time emergency recognition and alert notification, energy‐inefficiency and issues with clinical applicability, scalability and affordability of technological ‘solutions’ (Elger & Mormann, 2013; Gluckman & Schevon, 2015^*^).

Other articles emphasised the potential of online seizure diaries and detection devices to provide ‘accurate’ and ‘objective’ seizure reporting in a way that clinicians would see as useful: ‘Many [patients] do not track data at all, except in a general subjective ‘better, worse, about the same’ way. […] Written calendar data are difficult to transform into visually useful information depicting trends and relationships […] Technology can help, in the form of an electronic epilepsy diary’ (Fisher, 2010, p.516^*^). Little emphasis was placed on how technology mediates the detection and reporting process, or how it introduces different kinds of subjectivities that might require new ways of interpretation and negotiation by patients, families and clinicians. Articles on smartphone app technologies (e.g. Pandher & Bhullar, 2016; Ranganathan et al., 2015^*^) and treatment delivery implantable devices (e.g. Fisher, 2012; Nagaraj et al., 2015; Stacey & Litt, 2008^*^) also prioritised a technological focus and emphasised a view of the body as a site for technical intervention.

[Sensor] devices provide an exciting possibility of delivering an objective measurement scale to quantify the severity of seizure episodes […] Patients' or the care‐givers’ history regarding the severity of such events can be notoriously misleading, practically nullifying any meaningful derivations that can be made out of them. (Ranganathan et al., 2015, p.35^*^)

This view of technology as a tool assumes that success in improving clinical reporting and outcomes is largely an engineering task, rather than a sociotechnical one (in which, for example, shared understandings would co‐evolve with the technology). This foregrounds a view of technology as neutral with the potential to directly capture embodied experience when advanced enough to fulfil its clinical purpose. The body is seen as an object to be enhanced with new technology‐driven capacities (e.g. seizure reporting devices transmitting signal directly rather than requiring human input). People living with epilepsy and their support network are cast as passive technology users feeding the technology with complete and clinically useful data.

### Technology as platform

The lens of technology as *platform* refers not only to computing platforms in the technical sense (i.e. hardware and software that allow applications to run), but also to conceptual characteristics of technology‐supported programmes such as the ability to host content, facilitate exchange and invite contribution, all encompassed in a pre‐defined structure and parameters. This lens primarily underlies multi‐component studies of online patient education, self‐management and peer support. Although it engages more with the active role of human actors in self‐monitoring, its accounts remain deterministic in emphasising technology as directly capable of influencing knowledge levels and self‐management practices, and neglecting the role of patients and carers in co‐shaping (often collective) use and sensemaking within technology‐supported practices.

For example, the interactive WebEase self‐management programme included a seizure and triggers diary and behavioural change modules, along with a community discussion board and learning resources. Despite focused engagement with patients in a survey and two focus groups across different stages of iterative development, the programme was primarily evaluated on the basis of structured process and outcome measures. This included a small randomised controlled trial where self‐reported medication adherence and self‐efficacy were reported as increased (although the latter did not reach statistical significance) (DiIorio et al., 2011; DiIorio et al., 2009a^*^). It was not clear how the diversity of lived experiences was reflected in the online programme as the narrative was centred on the potential of the technology as a driver in transforming self‐management. Patient feedback as reported in this article was positive, although more work would be needed to achieve in‐depth understanding of real‐world use in the context of patient lives, with one patient commenting: ‘*I wanted an online journal to write down my feelings and thoughts about living with epilepsy and the program does not provide that’* (DiIorio et al., 2009a, p.193^*^). High levels of baseline medication adherence, self‐selected participation and short timeframes also meant that results may not be more widely generalisable or sustainable.

In another study, use of digital health tools in a Dutch group‐based self‐management programme proved challenging, as the smartphone application (seizure diary, medication reminder function and alarm mode) was withdrawn by the commercial provider and some patients found the medication monitoring system conflicted with their usual routine (Leenen et al., 2017^*^). What participants valued was ‘*peer‐ and social support, especially in sharing and comparing ideas about how to deal with the consequences of having epilepsy or having a relative with epilepsy’* with the authors concluding that ‘*research is still needed to establish whether using [the digital health solution] is the best way to give patients insight in their medication intake pattern*’ (Leenen et al., 2017, p.68^*^). Further studies articulated a platform view in their aspiration towards technology‐supported care, such as educational and risk assessment smartphone apps, but had not achieved sufficient integration with care practices or were criticised for inadequate information provision (Newman et al., 2016; Pandher & Bhullar, 2016; Shankar et al., 2015^*^).

### Technology as ‘intermediary’

Articles in this category primarily treated technology a) as a way to mediate certain types of patient care and clinical communication, often despite physical distance, that is, making patients and the condition manage‐*able* through technology and b) as a way to mobilise certain types of digital data generated by people living with epilepsy, that is, making patients and the condition know‐*able* by technological means.

In transcending physical distance, technologies included remote consultations (or telemedicine), wearable sensors and automated text messaging for remote management (Page et al., 2018; Patterson & Bingham, 2005; Rossi et al., 2015^*^). These studies focused largely on improving service efficiency and facilitating coordination between patients and specialists to increase the reach of limited clinical resources. Some of the sociotechnical complexity remained unarticulated, with one article suggesting that telemedicine is ‘*nothing more complicated than e‐mail for asynchronous communication and videoconferencing equipment*’ (Patterson & Bingham, 2005, p.614^*^) although more recent experience outside epilepsy shows routinisation of virtual consultations requires extensive effort, workarounds and displacements (Greenhalgh et al., 2019).

Studies on digital seizure diaries generally followed a logic of technology as an intermediary by which patients became better and more easily knowable to their clinicians. They emphasised the potential of electronic seizure diaries against traditional article‐based formats, including visual representations, standardised data, timely and accurate reporting, and automatic aggregation and analysis (Fisher, 2010; Fisher et al., 2015; Fisher et al., 2012; Pandher & Bhullar, 2016; Ranganathan et al., 2015^*^). Data from seizure diaries were often also harnessed for research on, for example, prevalence and frequency of cluster seizures (Fisher et al., 2015^*^), patient characteristics (Le et al., 2011^*^), medication and fertility (Ernst et al., 2016; French et al., 2016; Llewellyn et al., 2013; Pennell et al., 2012^*^) and stress and mood variables (Privitera et al., 2015^*^).

In focusing attention on easily quantifiable data, there is a risk that certain aspects of the condition would be prioritised, based on what is technically possible to collect and illustrate in visual formats. Seizure diaries alone are unlikely to provide the level of ‘accuracy’ sought, given patients are only aware of about half their daytime seizures (Blachut et al., 2015^*^). The information gained would be disembodied and partial, unless combined with other sources. More complex aspects of lived, embodied experience may be difficult to convey and communicate. We are not suggesting that technology drives this focus but that certain dimensions of technology tend to be foregrounded and mobilised specifically because they fit into a dominant, pre‐existing biomedical model relegating social aspects to the background. Rather than just looking at patients as recipients of technology‐enhanced ways of caring or as data providers, there may be scope to examine how technologies fit with their lives, their routines and ways of thinking about looking after themselves. Attention to the background care and information infrastructures that will need to accommodate emerging technologies at personal and organisational level is also needed (Greenhalgh et al., 2019).

### Attitudes to technology

Articles in this category approached the study of technology through ‘surrogate measures’ (Orlikowski & Iacono, 2001) including perceived acceptability, usability, uptake and satisfaction. Here, the technology constituted a fixed (and sometimes even imagined) artefact likely to either be accepted or resisted by patients, who were asked what technical features they would prefer and what attitudes they have towards certain technologies such as implantable devices (e.g. Patel et al., 2016; Arthurs et al., 2010*). Often user feedback was described as if it constituted rite of passage, without sufficient detail of how patient input had influenced development (with some exceptions, e.g. DiIorio et al., 2009b^*^). A large number of studies only surveyed prospective users, in some cases due to the technologies still being at experimental stage. Features assessed included sensor types, content options, ease of use and choice of interface (Kabir et al., 2015; Patel et al., 2016^*^).

Survey‐based methods dominated these studies, although samples remained small and timeframes limited. For example, Hixson et al suggested that almost half of participants in a six‐week trial of the epilepsy‐specific PatientsLikeMe platform reported better control over their condition and better understanding of their seizures (Hixson et al., 2015a; Hixson et al., 2015b^*^). Other studies branded their devices as ‘user‐friendly’ without explaining how this had been assessed (e.g. Beniczky et al., 2013; Bonnet et al., 2011^*^). The patient satisfaction survey as a ‘legitimising device’ was also common (Mort et al., 2009). Although epilepsy patients and caregivers generally reported favourable attitudes and interest in using digital health, actual uptake, usage and usefulness ratings remained low (DiIorio et al., 2009a; Leenen et al., 2016; Leenen et al., 2017; Liu et al., 2016; Newman et al., 2016^*^).

A disconnect existed between what prospective users (patients and health professionals alike) assumed the technology could do and actual technological capability. In focus groups, patients and health professionals expected wearables to transmit consistent ‘objective’ information and support ongoing communication with clinicians (Ozanne et al., 2017^*^). However, there are currently limitations in wearables being able to achieve consistent performance and regular patient–clinician communication may be difficult to sustain in a pressured health service. Patient views were sometimes described antagonistically (or in what could even be called symbolic violence), as in a study on brain‐implantable devices for seizure prediction and treatment (still at experimental stage) where it was recommended that research ‘*must overcome the reluctance of respondents to embrace implantation of devices*’ (Arthurs et al., 2010, p.477^*^).

### Sociotechnical studies

Referred to as ‘ensemble’ in Orlikowski and Iacono’s original taxonomy, this category would incorporate articles taking a broader, if not always social science, approach to the study of technology. It considers technologies as products of social (and political) processes and their development, use and non‐use as sociocultural acts. Articles in this category would imply (if not always explicitly consider), the social influences shaping (and being shaped by) technology, and would surface the complex relationships between technologies, patients, carers and clinical teams. In this sense, sociotechnical innovations are assumed to remain in formation, with users seen as playing a critical role in co‐shaping the technology in practice.

In the epilepsy literature, attention to sociotechnical aspects of innovation remained sparse. Social contexts of technology use entered the discussion mainly in relation to costs of information transmission, processing power and battery life in the context of busy user routines. Very few studies discussed technology as an ongoing sociotechnical accomplishment or considered early patient involvement in development (which of course brings its own complexities). One of the publications on the WebEase online self‐management programme included some reflection on the complexities of developing technology‐supported epilepsy care (DiIorio et al., 2009b^*^). In their lessons learnt, the authors raised communication challenges between development teams and the need for a common language:

The primary challenge was communication between Web programmers and content developers. […] To facilitate communication, we had several working sessions […] Communication was also facilitated by a team member with extensive experience in Web design who spoke the ‘language’ of both groups. (DiIorio et al., 2009b, p.4^*^)

A sociotechnical, participatory approach was mentioned in a conference abstract on patient access to electronic health records for epilepsy services in Ireland (Power et al., 2017^*^). Another article described an app introduced by the parent of a child with drug‐resistant epilepsy without, however, raising how lived experience contributed to development (Casassa et al., 2018^*^). Other studies on multi‐component interventions, including e‐health tools, decision support and peer networks for patients, could also be seen as encompassing a less deterministic view to the extent that the technology did not overly dominate ideas about how epilepsy care could be improved (Sajatovic et al., 2017; Shegog & Begley, 2015^*^).

Some articles offered historical, industry‐level perspectives on how engineered interventions for seizure prediction evolved over time (Gluckman & Schevon, 2015^*^); or focused on how industry‐wide standards and common data elements for mHealth in epilepsy could be developed (Goldenholz et al., 2018^*^). The team behind the EpSMon app for SUDEP risk assessment also provided an analysis of their trajectory from ‘concept to market’ following an innovation pathways approach (Newman et al., 2016^*^) although this perhaps imposed a more linear view than how innovations work in practice. With few exceptions, the literature on technology‐supported epilepsy care lacked in‐depth studies informed by a sociotechnical or other similar theoretical approach.

## DISCUSSION

Drawing on a hermeneutic analysis of the literature, we highlighted six lenses through which the role of technology has been treated in epilepsy care: computational artefact, clinical tool, platform, intermediary, reflected in user attitudes and (rarely) dynamic agent in sociotechnical systems (see Table 1). We developed this framework by interrogating how technology is positioned within published research and how (often unarticulated) assumptions about the ways in which technologies, patients and bodies are mutually shaped become reflected in articles’ research objectives, study designs and findings.

Our review shows that a highly deterministic, instrumental view of technology in epilepsy care dominates the literature. Patients are mainly cast as sources of attitudinal data, as beneficiaries of the technology or even as obstacles to be overcome. Properties of the technology are seen as inherent and become emphasised over views of technology as a sociotechnical accomplishment. This neglects the uncertainties, displacements and responsibilities that technology‐supported care generates. The logic of technology as dynamic agent in sociotechnical systems remains under‐developed, with nuanced, sociologically informed ways of engaging with patient‐facing applications largely missing.

With innovation viewed uncritically, there is little reflection on how assumptions about users are built into technologies, how they frame specific roles for users and their bodies, and how they privilege specific ways of patienthood (Mort et al., 2009; Prout, 1996). Despite the importance of family and professional carers in epilepsy, many of the technologies developed (e.g. wearables), only take patients as their primary locus of attention. Different responsibilities are being reproduced within each of these framings, echoing wider discussions about discourses of risk, individuality and responsibilisation (May et al., 2014; Prainsack, 2018; Rose, 2007). Epilepsy technologies are often designed with a view of patients as individually responsible to provide ‘good’ accounts of their self‐care, something that is assumed to be facilitated by the use of technology.

This prevalence of technological discourses is not surprising in the context of a condition where the brain is commonly paralleled to malfunctioning electrical circuits (Roach, 2017). Specific articulations of technologies imply particular subject positions, bodily accounts and relationships with future (and present) sociotechnical contexts. In these articulations, the future is ‘constituted through an unstable field of language, practice and materiality in which various disciplines, capacities and actors compete for the right to represent near and far term developments’ (Brown et al., 2000, p. 5). We believe it is important to articulate these imagined futures and interrogate the promise of technology, which is rarely realised without raising new concerns.

In this review, we followed Oudshoorn and Pinch (2003) in studying user representations as illustrated in the academic literature. The deterministic accounts we identified are tightly coupled with the conditions of their production, reflecting wider norms and professional visions that prioritise certain ways of engaging with innovation in academic and clinical research. Technological optimism is weaved into the accounts we analysed, perhaps driven by the need to justify research programmes supporting techno‐centric approaches and as a result of expectations raised and metrics applied in funding or investment competitions. Following taken‐for‐granted views about technology as a tool for efficiency and effectiveness within dominant techno‐economic rationalities, user representations that end up being taken in account are typically ‘interanimated by business concerns [i.e. economic] or technical possibilities/restrictions’ (Hyysalo & Johnson, 2016, p.86).

In this review, we have extended semiotic approaches to user research to highlight how user representations are not formed in isolation, but are inherently linked to and co‐shaped by representations of patient bodies and technological potentialities. We have offered a framework unpacking different ways of positioning technology and reflected on the implications of mobilising particular accounts and research designs. Social scientists can play a significant role in introducing different framings into technology‐supported epilepsy care (and other conditions); they can apply different lenses, techniques and methods that allow for a more theoretically informed set of viewpoints and values. Issues of digital inclusion (i.e. going beyond representations of what might be considered ‘typical’ users) appear especially neglected and primarily considered as an afterthought in technology development for epilepsy care. There is significant scope to expand research through greater attention to sociomaterial as well as affective and symbolic practices.

### Strengths and limitations

This is the first hermeneutic review on technology‐supported epilepsy care. We have supplemented structured systematic searching and screening with iterative ways of identifying and interpreting the literature. This allowed us to extend beyond a simple descriptive summary of existing studies, and to make sense of this complex, heterogeneous literature in critical and theoretically informed ways. There is a risk that our framework may be read in a deterministic manner, which is not our intention – rather our aim has been to map how the literature portrays current (and future) configurations between technology, users and patient bodies, and to propose ways forward for a more inclusive, sociologically informed study of technology‐supported epilepsy care. As in any interpretive process, there are limits to our analysis and possibilities that we did not consider. By bringing together a multidisciplinary team, we attempted to cover more ground than previous reviews and to increase methodological rigour. Our framework can also be used to interrogate literature in other long‐term conditions.

## CONCLUSIONS

Development of digital health ‘solutions’ in epilepsy care is moving fast. However, social studies that critically engage with this subject matter have not kept pace. In this article, we have highlighted the multiplicity of accounts, both competing and converging, that frame discussions on the role of technology in epilepsy care. They are largely enacting a particular techno‐scientific future for epilepsy care, while bracketing off other possibilities that would place more emphasis on the dynamic processes of co‐construction between users and technologies. Significant potential exists for social science contribution, bringing the study of epilepsy forward to the digital era.

## Author Contribution

**Chrysanthi Papoutsi:** Conceptualization (lead); Formal analysis (lead); Methodology (lead); Supervision (lead); Writing‐original draft (lead); Writing‐review & editing (lead). **Christian Collins:** Formal analysis (equal); Writing‐original draft (supporting). **Alexandra Christopher:** Formal analysis (supporting). **Sara Shaw:** Writing‐review & editing (equal). **Trish Greenhalgh:** Conceptualization (equal); Methodology (equal); Writing‐review & editing (equal).

## DATA AVAILABILITY STATEMENT

All reviewed papers are available on request

## Supporting information

Supplementary Material
