# Supplementary material for: Interrogating the promise of technology in epilepsy care: systematic, hermeneutic review
Source: Sociol Health Illn. 2021 Apr 1;43(4):928–47. doi: 10.1111/1467-9566.13266 (PMC8317050; doi:10.1111/1467-9566.13266)
Supplement: Supplementary file 1 — Supplementary Material [file SHIL-43-928-s001.docx]

# Supplementary sections

### Search strategy

**Search 1 – Example search Medline**

1. exp Epilepsy/
2. (epilep* or seizure?).ti,ab.
3. 1 or 2
4. monitoring, physiologic/ or monitoring, ambulatory/
5. Accelerometry/
6. Biosensing Techniques/
7. Electrodiagnosis/
8. (monitor* or device? or sensing or sensor? or track* or detect* or predict* or acceleromet* or manag*).ti.
9. (seizure? adj5 (monitor* or device? or sensing or sensor* or track* or detect* or predict* or acceleromet* or manag*)).ti,ab.
10. 4 or 5 or 6 or 7 or 8 or 9
11. Telemedicine/
12. Telemetry/
13. Online Systems/ or wireless technology/
14. cell phones/ or smartphone/ or microcomputers/ or computers, handheld/
15. (telemedicine or tele-medicine or telehealth or tele-health or ehealth or e-health or mhealth or m-health).ti,ab.
16. (online or on-line or virtual or electronic or digital or web* or internet* or realtime or real-time).ti.
17. ((online or on-line or virtual or electronic or digital or web* or internet*) adj5 (technolog* or system? or monitor* or device? or sens* or track* or detect* or predict* or manag*)).ti,ab.
18. wearable?.ti.
19. 11 or 12 or 13 or 14 or 15 or 16 or 17 or 18
20. 10 and 19
21. Remote Sensing Technology/
22. Mobile Applications/
23. (wireless or wifi or wi-fi or bluetooth*).ti,ab.
24. (smart adj (technolog* or monitor* or device? or watch* or track* or phone? or mobile? or home?)).ti,ab.
25. (smartwatch* or smartmonitor* or smarttrack* or smartphone? or smartmobile? or smarthome? or iphone? or i-phone? or ipad? or i-pad?).ti,ab.
26. (assist* adj2 living adj5 (technolog* or system? or monitor* or device? or sens*)).ti,ab.
27. (app or apps or mobile application?).ti,ab.
28. (((online or on-line or virtual or electronic or web* or internet) adj2 diar*) or e-diar* or ediar*).ti,ab.
29. (wearables or (wearable adj5 (technolog* or system? or device? or monitor* or sens* or track* or detect* or predict* or manag*))).ti,ab.
30. (implantable adj5 (technolog* or system? or monitor* or device? or sens* or track* or detect* or predict* or manag*)).ti,ab.
31. ((realtime or real-time) adj5 (technolog* or system? or monitor* or device? or sens* or track* or detect* or predict* or manag*)).ti,ab.
32. 20 or 21 or 22 or 23 or 24 or 25 or 26 or 27 or 28 or 29 or 30 or 31
33. 3 and 32
34. exp animals/ not humans.sh.
35. (rat or rats or mice or mouse or murine or rodent?).ti.
36. 34 or 35
37. 33 not 36
38. *Electroencephalography/
39. (eeg or eegs or electroencephalogra*).ti.
40. 38 or 39
41. 37 not 40

**Search 2 – Example search Medline**

1. exp Epilepsy/
2. (epilep* or seizure?).ti.
3. 1 or 2
4. exp *Patient Satisfaction/
5. *attitude/ or exp *attitude to health/
6. *adaptation, psychological/ or *emotional adjustment/
7. *Stress, Psychological/
8. help-seeking behavior/ or prejudice/ or social stigma/
9. catastrophization/ or optimism/ or pessimism/
10. exp Emotions/
11. ((patient* or health or illness) adj2 (experience? or expectation? or perspective? or perception? or preference? or view* or attitude? or opinion? or understand* or comprehen* or satisf* or account?)).ti,ab.
12. ((patient* or health or illness) and (experience? or expectation? or perspective? or perception? or preference? or view* or attitude? or opinion? or understand* or comprehen* or satisf* or account?)).ti.
13. ((epilep* or seizure?) adj5 (experience? or expectation? or perspective? or perception? or preference? or view* or attitude? or opinion? or understand* or comprehen* or satisf* or account?)).ti,ab.
14. ((epilep* or seizure?) and (experience? or expectation? or perspective? or perception? or preference? or view* or attitude? or opinion? or understand* or comprehen* or satisf* or account?)).ti.
15. ((psycholog* or mental) adj2 (stress or adapt* or adjust*)).ti,ab.
16. (feeling? or emotion*).ti,ab.
17. (cope or coping or "living with").ti,ab.
18. (stigma* or prejudice?).ti,ab.
19. 4 or 5 or 6 or 7 or 8 or 9 or 10 or 11 or 12 or 13 or 14 or 15 or 16 or 17 or 18
20. 3 and 19
21. exp Epilepsy/px [Psychology]
22. 20 or 21
23. (Qualitative systematic review* or (systematic review and qualitative)).ti,ab.
24. (evidence synthesis or realist synthesis or realist review).ti,ab.
25. (Qualitative and synthesis).ti,ab.
26. (meta-synthesis* or meta synthesis* or metasynthesis).ti,ab.
27. (meta-ethnograph* or metaethnograph* or meta ethnograph*).ti,ab.
28. (meta-study or metastudy or meta study).ti,ab.
29. systematic review*.ti,ab. and qualitative research/
30. 23 or 24 or 25 or 26 or 27 or 28 or 29
31. qualitative research/
32. *interviews as topic/ or focus groups/ or narration/
33. observation.ti.
34. interview?.ti.
35. (qualitative adj2 (interview* or study or research)).ti,ab.
36. qualitative.ti.
37. (focus group? or story or stories or narration or narrative* or discourse or discursive or grounded theory or ethnogra* or phenomenolog*).ti,ab.
38. 31 or 32 or 33 or 34 or 35 or 36 or 37
39. 22 and 30
40. 22 and 38
41. 39 or 40
42. limit 41 to (english language and yr="2007 -Current")

### Descriptive study details

Table 1: Study characteristics of 111 high relevance articles resulting from original search 1 (epilepsy and technology) and citation tracked

|  | **Name** | **Year** | **Country** | **Type of paper** | **Study design/methods** | **Sample/setting** | **Objectives** | |
| --- | --- | --- | --- | --- | --- | --- | --- | --- |
|  | Arthurs et al | 2010 | USA | Research | Survey questionnaire made available online or in printed form. | 89 responses were received from 56 patients with epilepsy and 33 family caregivers. | To elicit patient and carer views on issues such as epilepsy treatment, seizure prediction and implantable devices. | |
|  | Beniczky et al | 2013 | Denmark | Research | Observations and quantitative measurement of seizure detection accuracy. | 73 patients (39 male; age 6–68 years, median 37 years) at risk of having generalised tonic clonic seizures, admitted to long-term video-EEG monitoring in three epilepsy centres; 20 of them recorded seizures during the study period. | To assess the clinical reliability of a wrist-worn, wireless accelerometer sensor for detecting generalized tonic clonic seizures. | |
|  | Bidwell et al | 2015 | USA | Research | Expert opinion, descriptive literature review and online questionnaire to assess clinical information needs for epilepsy treatment, followed by a descriptive review of seizure detection and classification technologies. | 10 clinicians specialising in clinical care of people with epilepsy. | To assess clinical information needs for epilepsy treatment and review the performance of current seizure detection and classification technologies, compared to patient self-reporting. | |
|  | Blachut et al | 2015 | Germany | Research | Survey questionnaire (postal) | 174 adult patients with epilepsy who had experienced seizures in the past year responded to the survey. | To examine the practice of keeping seizure diaries and the patients’ attitudes toward seizure counting. | |
|  | Bonato | 2010 | USA | Review/discussion paper | Descriptive review. | Not specified. | To provide a summary of wearable technologies suitable for clinical applications (including epilepsy). | |
|  | Bonnet et al | 2011 | France | Research | To present the architecture of a remote monitoring system for seizure detection. | Preliminary algorithm testing with 2 people with epilepsy (no other details provided) in home environment. | To present a remote monitoring system for young people with epilepsy to detect night time seizures using body worn sensors. | |
|  | Borujeny et al | 2013 | Iran | Research | Algorithm development. | Datasets from 3 patients living with severe epilepsy for the development of an automatic detection algorithm. | To present and evaluate a seizure detection system based on accelerometry for the detection of epileptic seizures. | |
|  | Carlson et al | 2009 | USA | Research | Efficacy study of a bed seizure monitor to detect generalized tonic-clonic seizures. | 64 in-patients (13–65 years) in a hospital-based video electroencephalography unit (1528 total hours of monitoring). | To investigate the sensitivity and specificity of the Medpage bed seizure monitor. | |
|  | Cavazos et al  (abstract) | 2015 | USA | Research | Phase III double-blind controlled device trial at 11 epilepsy monitoring units. | Electromyography and video- electroencephalography data recorded from 157 in-patients with history of generalised tonic clonic seizures. | To validate the effectiveness of a novel electromyography-based, real-time, generalised tonic clonic seizure detection system that can be worn on the body. | |
|  | Cogan et al | 2017 | USA | Research | Experimental study to test the accuracy of a seizure detection algorithm. | Dataset collected through video electroencephalography from 10 patients admitted to an epilepsy monitoring unit  . | To develop and test the accuracy of the algorithm. | |
|  | Conradsen et al | 2012 | Denmark | Research | Experimental study to assess the performance of a seizure detection device. | 5 patients (15-48 years old) from the Danish Epilepsy Center. All had a history of generalised tonic clonic seizures and were admitted for 2-5 days. | To present the results of an implementation of a novel algorithm into a wireless detection device | |
|  | Cook et al | 2013 | Australia | Research | Multicentre, first-in-man clinical feasibility study, including surgical implantation of devices for seizure prediction and individualised algorithms. | 15 ambulatory patients with drug-resistant epilepsy recruited from three epilepsy centres. | To assess the safety and efficacy of a long-term implanted seizure advisory system designed to predict seizure likelihood and quantify seizures in adults with drug resistant focal seizures. | |
|  | Cuppens et al | 2014 | Belgium | Research | Technical and algorithm development and validation. Seizures were monitored overnight using accelerometers attached to extremities. | 7 patients with hyper-motor seizures (5-16 years). | To propose a method for hypermotor seizure detection based on accelerometers attached to the extremities. | |
|  | de la Loge et al | 2016 | Belgium | Research | Retrospective quantitative analysis based on data collected on the online PatientsLikeMe Epilepsy Community. | PatientsLikeMe Epilepsy Community members’ data including demographic, disease, and treatment characteristics. | To characterize the profile of users and their disease and identify factors predictive of poor health-related quality of life, while assessing the platform's potential in providing patient-reported data for research purposes. | |
|  | DiIorio et al | 2009b | USA | Conceptual paper | Overview of the online self-management WebEase programme which involved 3 modules on medication, sleep and stress management, a daily log, discussion boards, fact sheets, quizzes, daily poll questions, and links to online resources. | Development comprised multiple stages, including a literature review, computer use survey, a focus group with 6 people with epilepsy, review by content experts and consumers, and a pilot study (with 37 participants). | To present the intervention components and the different stages of their development as part of the WebEase programme including reflection on lessons learned. | |
|  | DiIorio et al | 2009a | USA | Research | Feasibility and acceptability study of WebEase, an Internet based, theory-driven, self-management program for adults with epilepsy. In this 6-week pilot implementation participants completed psychometrically validated before and after measures on epilepsy self-management, self-report adherence, stress, sleep quality, epilepsy knowledge, personal resource, self-efficacy, outcome expectancies, as well as usability and satisfaction. | 35 people living with epilepsy (mean age 37.5 years) recruited from two hospital-based clinics. | To assess the feasibility, acceptability, usability and behavioural outcomes of WebEase. | |
|  | DiIorio et al | 2011 | USA | Research | Experimental study on a 6-week online epilepsy self-management program (WebEase). Those randomized to the treatment group received the program first, whereas waitlist control participants started 6 weeks later. | 148 adult living with epilepsy (18-77 years old). | To determine if the online WebEase self-management programme could support participants with improvements in medication adherence, perceived stress, and sleep quality. | |
|  | Dourado et al  (abstract) | 2009 | Portugal | Research | The project uses multi-signal information, intelligent data processing and wireless communications. It involves data analysis, algorithm development, and knowledge development around technologies. These components integrate into an intelligent system. An epilepsy database will also be developed, containing participants’ data which can be used for semantic mining based on multimodal, multi-signal and multi-dimensional data. | Not adequately specified in this abstract although there is reference to datasets from epilepsy patients in three participating hospitals. | To present plans for developing a portable, intelligent alarm system, which measures brain activity and predicts seizures. | |
|  | Dourado et al | 2014 | Portugal | Research | The project includes a number of different workpackages: a) development of the Epilepsy Database, b) the EPILAB computational framework for training and testing seizure prediction algorithms, c) the Brainatic seizure prediction device and testing its performance on the Epilepsy Database using machine learning algorithms. | This multicomponent study developed a European epilepsy database drawing on long term acquired multichannel EEG data from 275 patients in Germany, Portugal and France. The seizure prediction device was also tested using data from 278 patients. Data containing 1519 seizures were used to test whether the algorithm accurately classified each feature. | To present the results of the FP7 Project Epilepsiae, including the computational framework EPILAB, The European Epilepsy Database, and the Brainatic prototype of a seizure prediction device for use by refractory epilepsy patients. | |
|  | Elger & Mormann | 2013 | Germany | Commentary | n/a | n/a | To highlight the Cook et al (Cook et al., 2013) study as a major milestone in epileptology showing for the first time that prospective seizure prediction is possible. | |
|  | Ernst et al | 2016 | USA | Research | Quantitative analysis as part of a prospective multi-center observational study to evaluate fertility in women with epilepsy versus age-matched controls. Participants were provided with the WEPOD app to track menstrual cycle, sexual activity, seizures and antiepileptic drugs. | 86 women with epilepsy who were planning pregnancy. | To examine medication adherence among women with epilepsy via use of an electronic diary. | |
|  | Fisher | 2010 | USA | Commentary | n/a | n/a | To discuss the importance of seizure tracking, challenges in effectively achieving this, and the potential of Epilepsy.com and ‘My Epilepsy Diary” in supporting monitoring. | |
|  | Fisher | 2012 | USA | Review | Descriptive presentation of clinical evidence | n/a | To summarise selected therapeutic neuromodulation devices and their potential to be used in treatment for epilepsy. | |
|  | Fisher et al | 2012 | USA | Review | Descriptive literature review and expert opinion | n/a | To summarise opportunities and challenges with using electronic seizure diaries. | |
|  | Fisher et al | 2015 | USA | Research | Observational study quantitatively analysing descriptive longitudinal data from electronic seizure diaries. | Anonymised data downloaded from 28,697 user profiles on the My Epilepsy Diary. | To assess seizure patterns based on self-reported data from an online seizure diary. | |
|  | French et al  (abstract) | 2016 | USA | Research | Multi-centre prospective, observational study of women with epilepsy and healthy controls | 88 women with epilepsy and 109 healthy controls aged 18-41 and planning pregnancy. | To compare time to pregnancy and outcomes (live birth, miscarriage) among women with epilepsy and healthy controls. | |
|  | Frings et al | 2008 | Germany | Research | Pilot study: questionnaires and cognitive tests were completed by participants on an electronic device over 6 days to compare cognition, psychophysical conditions, aggressiveness and concentration between those receiving add-on treatment with levetiracetam and controls without add-on treatment. | 20 adult patients with epilepsy were recruited by the outpatient clinic. | To report the application of a new device for temporally fine-grained assessment of objective well-being and cognitive performance using personal digital assistants. | |
|  | Ge et al  (abstract) | 2017 | USA | Research | Observational study using descriptive statistics to assess seizure triggers as reported by users of the EpiWatch app. | 598 patients enrolled in the EpiWatch study during an initial 10-month study period | To identify common seizure triggers in a US epilepsy population using the EpiWatch research app. | |
|  | Gibney | 2015 | UK | Commentary | n/a | n/a | Discusses the potential of implantable or skin-mounted sensors to harvest biomedical data on diseases, such as epilepsy, and challenges including how to power such devices and potential for external compromise (hacking). | |
|  | Gluckman & Schevon | 2015 | USA | Editorial | n/a | n/a | To provide a short history of seizure prediction, outline significant achievements and outline the role of the International Seizure Prediction Group. | |
|  | Goldenholz et al | 2018 | USA | Research/ consensus development | Consensus development between key stakeholders in industry, patient advocacy groups, and clinician scientists. | Meeting and subsequent communication with a consortium of key stakeholders, leading to the development of a consensus statement on back-end data collection by epilepsy devices and apps. | To develop an approach to industry-wide common data elements for m-health epilepsy technologies. | |
|  | Gouravajhala et al | 2012 | USA | Research (technical development) | The paper describes the technical system implementation and covers initial findings from testing the app on an emulator. | n/a | To describe the technical and algorithmic components of "EpSMART: Epileptic Seizure  Monitoring with Alerts in Real Time" a tablet-based Android application for a multi-modal seizure detection system, currently in-progress. | |
|  | Gubbi et al | 2016 | Australia | Research (algorithm development) | The paper describes a wrist-worn accelerometer sensor and the development and testing of a classification algorithm for convulsive seizures. | Dataset collected from 27 inpatients who experienced motor seizures during hospitalization in an epilepsy unit. | To develop a wearable seizure detection system and classification algorithm that distinguishes epileptic from non-epileptic seizures. | |
|  | Hall et al  (abstract) | 2009a | USA | Research | Observational study using prospective data collection of symptom data on electronic diaries. | 19 patients with a history of 3+ seizures per month. | To report associations between premonitory  symptoms and short term seizure incidence. | |
|  | Hall et al | 2009b | USA | Research | Modelling approaches used to predict the occurrence of future seizures based on self-reported diary data. | 71 adults living with localisation-related epilepsy (65% female, median age 41.1) collected 30 days’ nightly seizure data and potential seizure precipitants. | To develop a model using monitoring data to predict subsequent seizures. | |
|  | Harper et al  (abstract) | 2016 | USA | Research | Online physician satisfaction survey administered to community-based hospital physicians via REDCap, analysed using descriptive statistics. | 15 physicians at nine community hospitals completed 68 physician surveys following teleneurology consultations. | To describe a teleneurology consultation service for nine community-based hospitals and to elicit feedback from physicians. | |
|  | Haut | 2013 | USA | Commentary | n/a | n/a | To discuss the study by Cook et al (Cook et al., 2013) highlighting the potential to predict seizures with high sensitivity but also pointing to the adverse events identified and questions around clinical seizures against seizures as experienced by patients. | |
|  | Haut et al | 2013 | USA | Research | Observational study where patients maintained an e-diary twice-daily reporting mood data, premonitory symptoms, and all seizures. | 19 adults patients with localisation-related epilepsy and ≥3 seizures per month (median age 35 years). | To use an e-diary with time-stamped data to ensure prediction precedes seizures, and to characterize prodromal features and time windows that underlie self-prediction. | |
|  | Haut et al | 2012 | USA | Research | Observational study where patients maintained an e-diary twice-daily reporting mood data, premonitory symptoms, and all seizures. Analysis using statistical univariate and multivariate models. | 19 adults patients with localisation-related epilepsy and ≥3 seizures per month (median age 35 years). | To use an e-diary with time-stamped data to clinically characterise the pre-ictal state in terms of premonitory features. | |
|  | Heldberg et al | 2015 | Germany | Research | Algorithm development and testing using a hierarchical classification approach to detect different types of epileptic seizures using electrodermal activity data and motion measuring acceleration from wearable sensors. | Dataset collected from wearable sensors worn by 8 inpatients diagnosed with epilepsy and scheduled for a video-electroencephalography monitoring at an epilepsy centre (the dataset included 55 epileptic seizures in 540hrs of recording). | To use electrodermal activity and motion measuring acceleration to try and detect seizures using wearable sensors and to determine significant characteristics and differences between seizure types. | |
|  | Helmy and Helmy | 2015 | USA | Research | Algorithm implementation and refinement to improve sensitivity and specificity. The Seizario smartphone uses accelerometer-based learning algorithms to automatically detect seizures and falls. | Device testing followed by testing with simulated seizures/falls from 14 volunteers in experimental settings. There is also mention of algorithms applied to data ‘from the field’ with the app running for 20 weeks during daily activities (but little detail is provided). | To present the Seizario app architectural overview and performance testing, and to compare its potential against other technologies for seizure detection. | |
|  | Hixson  (abstract) | 2016 | USA | Research | Validated self-efficacy and self-management metrics before and after using the platform. | 249 US veterans diagnosed with epilepsy (mean age 50.2) joined the online platform and 92 completed 6 weeks of participation. | To test the effectiveness of an online patient community to improve epilepsy self-management. | |
|  | Hixson et al | 2015c | USA | Research | Pre-/post metrics for patient self-management and self-efficacy, patient satisfaction survey and usage data. Analyses were based on both completer and intention-to-treat scenarios. | 249 US veterans diagnosed with epilepsy (mean age 50.2) joined the online platform and 92 completed 6 weeks of participation. | To test whether engaging in an online patient community  improves self-management and self-efficacy in veterans with epilepsy. | |
|  | Hixson et al  (abstract) | 2015a | USA | Research | Pre-/post metrics for patient self-management and self-efficacy, patient satisfaction survey and usage data. | 249 US veterans diagnosed with epilepsy (mean age 50.2) joined the online platform and 92 completed 6 weeks of participation. | To assess participant usage of a digital health platform and (as stated in the paper) qualitative satisfaction with the experience (although no qualitative methods are reported). | |
|  | Hixson et al | 2015b | USA | Research | Quantitative, descriptive usage statistics and retention analysis. | 249 US veterans diagnosed with epilepsy (mean age 50.2) joined the online platform and 92 completed 6 weeks of participation. | To describe user retention and site activity for three months following completion of a study using a digital health platform to improve self-management. | |
|  | Horizon Scanning Research & Intelligence Centre | 2017 | UK | Report | Descriptive review and expert/patient input | n/a | To identify emerging technologies for the diagnosis and management (including treatment) of epilepsy. | |
|  | Jones et al | 2008 | The Netherlands | Research/  technical description | Description of the components and technical configuration of a technological solution. | n/a | To describe an application which processes and analyses biosignals and context information, including positioning of patient and carers, to identify medical emergencies and facilitate a response. | |
|  | Jory et al | 2016 | UK | Systematic review | Systematic search in 3 databases and screening following PRISMA guidelines. . | At least 3 of the following criteria: use of control cases, confirmed diagnosis, 10 or more cases, identification of false positives and/or quality of life mentioned. Additional papers were used to provide context. | To systematically review the literature on seizure detection devices and assess the quality of papers retrieved. | |
|  | Kabir et al  (abstract) | 2015 | USA | Research | Qualitative (focus groups) analysed thematically | 2 focus groups with total 11 people living with epilepsy (mean age 54). | To assess acceptance of a mobile telecare system (e.g. video consultations) to support self-management for people living with epilepsy and to identify possible issues for further improvement. | |
|  | Kingwell | 2013 | n/a | Commentary | n/a | n/a | To present the study by Cook et al. (see (Cook et al., 2013)) and discuss its key findings including the high number of adverse reactions, and discrepancy between actual and reported seizures. | |
|  | Kramer et al | 2011 | Israel and USA | Research | Pilot study to test an accelerometer device and algorithm for seizure detection. | 31 adult patients from inpatient video EEG units in two epilepsy centres.  Included patients with a history of primarily motor seizures including clonic, tonic, partial, or generalized and patients with complex partial seizures who had generalized seizures after tapering of antiepileptic drugs as part of the monitoring. | To describe the preliminary results of testing a portable wearable accelerometer and algorithm software to detect seizures. | |
|  | Le et al | 2011 | USA | Research | Retrospective, descriptive study on the user database of the My Epilepsy Diary. | Users of the My Epilepsy Diary (average age 29, 64% women) during 2010. Total of 1944 patients submitted demographic data. | To outline electronic diary functions and retrospectively profile users including demographics, seizure types, temporal distribution of seizures, triggers, use and side effects of antiepileptic drugs. | |
|  | Leenen et al | 2016 | The Netherlands | Research | Survey questionnaire, including open-ended questions. | 571 respondents (259 adults living with epilepsy, and the rest of respondents were primarily parents and caregivers). Average age 38.3 years. | To explore whether people living with epilepsy have access to digital health tools and if they perceive them to be useable for their care. | |
|  | Leenen et al | 2017 | The Netherlands | Research | Prospective mixed methods study. | 52 patients with epilepsy living in community/home settings, 37 relatives and 6 facilitators. Data gathered using questionnaires for patients and relatives, registration forms for facilitators and by carrying out semi-structured group interviews with patients, relatives and facilitators. | To establish the feasibility, fidelity and acceptability of a multi-component self-management intervention for patients and their relatives. | |
|  | Lehnertz | 2001 | Germany | Review | Descriptive review. | n/a | To give an overview of the state of the art of seizure anticipation techniques and future prospects for seizure anticipation devices**.** | |
|  | Litt and Echauz | 2002 | USA | Review | Descriptive review. | n/a | To review the literature on epileptic seizures prediction: its history, current state and future trends including the need for high quality datasets of intracranial recordings and clinical trials, and momentum towards intelligent seizure treatment devices. |  |
|  | Litt | 2001 | USA | Review | Descriptive review. | n/a | To discuss the potential for devices that can detect seizures and offer therapeutic interventions for people with epilepsy. |  |
|  | Liu et al | 2016a | China | Research | Cross-sectional study using survey questionnaire as data collection method. | 390 caregivers of children living with epilepsy, recruited in an inpatient unit (mean age of 36.6 years). | To determine the feasibility as well as the attitudes among caregivers of children and adolescents with epilepsy in China towards the use of smart phone applications for the management of seizures. |  |
|  | Liu et al | 2016b | China | Research | Cross-sectional study using a survey questionnaire | 502 people living with epilepsy recruited from an epilepsy unit (mean age 27.98 years old). | To assess feasibility and acceptability of smartphone apps for seizure self-management. |  |
|  | Llewellyn et al  (poster) | 2013 | USA | Research | Interim analysis from a 3-site prospective, observational study evaluating fertility in women with epilepsy. | 119 women with epilepsy and healthy controls enrolled in the study (mean age 31.3 years). Participants recorded menstrual bleeding and sexual activity daily and tracked seizure occurrence and medication compliance. | To assess use of the WEPOD (Woman with epilepsy: pregnancy outcomes and deliveries) app and whether data entry fatigue occurred over the course of participation. |  |
|  | Lockman et al | 2011 | USA | Research | Proof in principle study for seizure detection technology development. | 40 patients (23-48 years) were monitored using the device in an inpatient setting. | To evaluate a wrist-worn motion detection device (The SmartWatch) to ascertain if it would detect convulsive seizure movements early in the course of the seizure. |  |
|  | Maiwald et al | 2011 | Germany | Research | Prospective observational study, using personal digital assistants. | 11 people living with focal or generalised epilepsy agreed to participate in the study (mean age 39 years, range 20–52 years, 9 females) and 9 of them returned data after a 4-5 week period using the device in the community. | To develop a methodology for prospectively assessing sensitivity and specificity of sensations that occur prior to seizures, using personal digital assistants. |  |
|  | Mormann et al | 2007 | USA | Review | Descriptive review | n/a | Discussing and reviewing technological problems with predicting seizures from EEG recordings and the problems and pitfalls with seizure prediction algorithms. |  |
|  | Morell  (abstract) | 2009 | USA | Research | Multicentre double blind randomized controlled trial on the efficacy and safety of the RNS Neuropace implantable. | 181 patients with retractable epilepsy having on average 3 seizures per month, across 3 sites. | Evaluation of safety and efficacy of cranially implanted response stimulator. |  |
|  | Myers et al | 2016 | USA | Research/ technical architecture development | Algorithm development | n/a | To develop an algorithm for an ambulatory seizure monitoring system |  |
|  | Nagaraj et al | 2015 | USA | Review | Descriptive review | n/a | Discussion of algorithms to predict seizures and hardware used to implement these technologies. |  |
|  | Newman et al | 2016 | UK | Research/ technology development | Descriptive overview of design, development and implementation for the Epilepsy Self-Monitor (EpSMon) smartphone-based app. | 221 patients/users downloaded the technology in the first three months – number considered low so the paper focuses on changes needed to the model of delivery and marketing. No other information on current users is provided. | To present the development and implementation of an eHealth tool to support education and risk management in epilepsy. |  |
|  | Nijsen et al | 2005 | The Netherlands | Research | Comparative assessment of seizure detection methods. | Database containing 36 hours of EEG recordings and 5 days of video monitoring, 3D accelerometry recordings and ECG recordings of 18 patients with severe epilepsy (mean age 37 years) in long-term care settings. | Assessing the validity of 3D accelerometer in seizure detection in patients with severe epilepsy |  |
|  | Ozanne et al | 2017 | Sweden | Research | Qualitative focus group study. | Three focus groups involving 10 people living with epilepsy (average age 36), and one focus group with 7 health professionals working in epilepsy. Separate focus groups were carried for people living with Parkinson’s and service providers. | To explore perceptions regarding the use of wearable technology in disease monitoring and management as reported by individuals with epilepsy and Parkinson’s disease as well as health professionals working with these patient groups. |  |
|  | Page et al  (abstract) | 2016 | UK | Research | Algorithm development for seizure detection. | Seizure detection data from inpatients and outpatients over 4 months (no other details provided). | To describe a combination of technologies piloted to detect seizures in patients with epilepsy. |  |
|  | Page et al | 2018c | UK | Conceptual | Description of a technology-supported epilepsy service, including opportunities for care improvement and potential challenges. | n/a | To discuss a conceptual framework for the use of technology in the epilepsy service. |  |
|  | Pandher et al | 2016 | Australia | Review | Descriptive overview of applications used to manage seizures. | n/a | To examine salient features of smartphone applications for seizure management. |  |
|  | Patel et al  (extended abstract) | 2009 | USA | Research | Preliminary results from the evaluation of algorithms for seizure detection, drawing on accelerometer and electromyography data. | Data collected from 5 patients over 2-5 days in an inpatient setting. Results presented for one patient. | To present preliminary results (implementation process and analysis outcomes for one patient) from an ongoing study on wearable sensors for seizure detection. |  |
|  | Patel et al | 2016 | USA | Research | Quantitative research using a survey questionnaire and descriptive statistics. | 904 patients with epilepsy or caregivers completed the survey. | To identify seizure detection device features that are important to patients living with epilepsy and their caregivers. |  |
|  | Patterson et al | 2015 | USA | Research | Prospective study testing a wrist-worn seizure detection monitor, and comparing to other seizure detection systems. | 41 patients (ages 5-41 years) participated in the study which took place an epilepsy monitoring unit at a tertiary hospital. | To assess the sensitivity and reliability of a commercially available wrist-worn monitor (SmartWatch) to detect various seizure types. |  |
|  | Patterson and Bingham | 2005 | Australia | Editorial | n/a | n/a | Discusses potential role for telemedicine in epilepsy (e.g. remote consultations). |  |
|  | Pennell et al  (abstract) | 2012 | USA | Research | Interim descriptive analysis as part of a prospective, observational study across three sites. | 16 women with epilepsy and 21 controls, aged between 18 and 40 years, from the WEPOD cohort (women with epilepsy: pregnancy outcomes and deliveries). | To assess feasibility and timeliness of data entry on a smartphone diary app for women with epilepsy (WEPOD app). |  |
|  | Picard et al | 2017 | USA | Case report | Case report | Single patient with SUDEP. | To present a case of a wrist sensor detecting changes in physiology before SUDEP. |  |
|  | Poh et al | 2012 | USA | Research | Development and testing of seizure detection algorithm for a wrist-worn sensor. | 80 patients admitted to a long-term video-EEG monitoring unit were included in the study but only 7 patients contributed generalised tonic clonic seizure data for the dataset collected. | To evaluate algorithm and sensor (accelerometer and electrodermal activity) performance for the detection of convulsive seizures. |  |
|  | Polak et al  (abstract) | 2014 | USA | Research | Prospective observational study comparing daily diary adherence over 8 weeks across three methods: 1) paper diary 2) e-diary with manual upload of data via a telephone server and 3) data from an early phase clinical trial of a smartphone based e-diary. | Adults living with focal epilepsy in home/community settings. | To determine whether electronic diaries increase adherence with data entry compared to paper diaries. |  |
|  | Popescu et al  (abstract) | 2013 | Romania | Research /device description | Describes a device attached to the neck of the patient using a necklace type grip, to detects and decode brain signals specific to epileptic seizures, and warn the patient, their carers, emergency and other clinical services. | Not specified in the abstract | To describe a device for real-time monitoring of epileptic seizures and early notification of impending seizure. |  |
|  | Poppel et al | 2013 | USA | Research | Prospective comparative study | 45 children and young adults (14 months to 28 years) who were inpatients in an epilepsy monitoring unit. | To test the sensitivity and specificity of the Emfit movement monitor (considered the best on the market) in detecting a variety of seizures in children and young adults overnight, comparing performance with standard video EEG technology, cardiopulmonary and nursing monitoring. |  |
|  | Privitera et al  (abstract) | 2015 | USA | Research | Prospective RCT which compares stress reduction exercises to sham treatments using a smart phone to record stress and mood levels. | 67 patients in 3 epilepsy centres were randomised to relaxation exercises vs sham treatments. | To evaluate whether stress reduction exercises reduce seizure frequency, using smartphone diary monitoring of stress and mood variables. |  |
|  | Raam and Sasikala | 2016 | India | Review/ proposed system architecture | Descriptive review of the literature on seizure detection and prediction, including product review, followed by basic description of the system architecture of a new wrist-worn device. | n/a | To propose the design, development and validation of a wrist-based wearable device. |  |
|  | Rangathan et al | 2015 | India | Review | Descriptive review | n/a | To provide an overview of how mobile phones can be used as diagnostic, prognostic or therapeutic tools in epilepsy. |  |
|  | Reeder and David | 2016 | USA | Review | A systematic review of smart watch studies that engaged people in their use. Participant demographics, device features, watch applications and methods, and technical challenges were extracted from included studies. | Inclusion criteria were English-language articles or conference papers that reported use of smart watch technology for health or wellness-related purposes. Exclusion criteria were studies that described proposed designs, architectures, frameworks, and models; poster and meeting abstracts; and workplace and driving safety applications | To characterize the application of smart watch devices in health-related research and identify methodological issues related to the use of smart watches for support of health and wellness in everyday life. |  |
|  | Regalia et al  (conference poster) | 2015 | USA | Research | Comparative study training and testing the performance of different seizure detectors. | Recordings consisted of video electroencephalography, electrodermal activity and wrist acceleration from 9 patients wearing a wrist device. | To improve automated seizure detection systems by combining wrist acceleration and electrodermal activity. |  |
|  | Roach | 2017 | UK | Research | Critical analysis and discussion of epilepsy discourses underpinned by qualitative research. | Drawing on 15 semi-structured interviews conducted with members of the Epilepsy Action e-mail list between May 2014 and December 2015. | Critically discussing how epilepsy is situated within new media and contemporary technological discourses. |  |
|  | Rodriguez-Villegas et al  (abstract) | 2009 | UK | Research/ technical development | Device development | Not stated | To develop a device that can detect apnoea in order to reduce risk of SUDEP. |  |
|  | Rossi et al  (abstract) | 2015 | USA | Research/ service improvement | Implementation of an intervention with 5 sub-components (video-conferencing communication protocol, wearable ambulatory body sensor suit technology, custom-designed web-based networking technology, computer based education series targeting epilepsy and mental health and an independent community based hub which facilitates the above 4 components). | Those living rurally with refractory epilepsy. | To develop and implement a novel patient-centered population health management (PHM) outreach delivery model that capitalises on mobile health information technologies for chronic epilepsy care. |  |
|  | Sajatovic et al | 2017 | USA | Report | Range of approaches described for research and service improvement. | Various studies including different participants although little detail provided in this paper. | To provide an update on the Managing Epilepsy Well (MEW) Network research pipeline, which spans efficacy, effectiveness, and dissemination. The authors outline (briefly) existing interventions and those in the pipeline, some of which involve technology. |  |
|  | Salem et al | 2014 | France | Research | Algorithm development. Performance of the algorithm was evaluated using a synthetic data set. | Data from one patient with epilepsy was collected from accelerometers in an experimental setting (no further information about the patient provided). | To provide a lightweight  approach for early detection of nocturnal epileptic seizures  using data from wireless 3-D accelerometer sensors. |  |
|  | Sareen et al | 2016 | India | Research | Development of a seizure detection model, including algorithms for uploading and storing patient data, generating alert signals, analysing and classifying electroencephalography signals. | Publicly available dataset from another study, measured by using 14 surface electrodes placed on the skull. | To propose a mobile-based framework that automatically predict seizures using information contained in electroencephalography signals. |  |
|  | Schwartz | 2007 | USA | Review | Descriptive review of clinical evidence | Not specified. | To evaluate haemodynamic markers with brain imaging modalities of SPECT , fMRI, PET and their potential in seizure prediction |  |
|  | Serhani et al | 2016 | UAE | Research | Description and testing of technical architecture | Only evaluated using experimental scenarios and existing datasets in epilepsy. | To develop a smart mobile end-to-end monitoring architecture to monitor and visualise life-long diseases. |  |
|  | Shankar et al | 2015 | UK | Editorial | Discussing the important of managing risk in epilepsy especially in relation to Sudden Unexplained Death in Epilepsy. | n/a | To describe the development of EpSmon, an app that aims to supports service users to monitor their own risk. |  |
|  | Shegog and Begley  (abstract) | 2015 | USA | Research | Randomised controlled trial to compare MINDSET to a usual care only scenario. Outcomes measured the impact of MINDSET on patient self-management. | 42 adult patients at three participating neurology specialty clinics. | To describe the development and evaluation of the Management Information & Decision Support Epilepsy Tool (MINDSET). |  |
|  | Shegog et al | 2013 | USA | Review/ commentary | Descriptive overview of different Managing Epilepsy Well (MEW) interventions including e-Tools (the MEW website, WebEase, UPLIFT, MINDSET, and PEARLS online training). | People living with epilepsy | To describe ongoing research by the Managing Epilepsy Well (MEW) Network and its partners that investigates the potential of e-Health to provide self-management support to people with epilepsy |  |
|  | Stacey and Litt | 2008 | USA | Review | Descriptive review | Not specified | To describe cutting-edge strategies for devices to control epilepsy and highlight promising areas that are under active investigation. |  |
|  | Stigwall et al  (abstract) | 2008 | Sweden | Research | Prospective study. Motion data was recorded using wireless acceleration sensors. EEG recordings were used as a reference. Patient feedback was also sought. | 38 patients whose motion data was recorded during hospital-based seizure monitoring (200 seizures total over 131 days). | To demonstrate sensitivity and specificity of a seizure logging technology using accelerometry to quantify number of seizures. |  |
|  | Sun et al | 2014 | USA | Review | Descriptive review that presents the history of the development of four closed-loop systems, which are systems that modulate or adapt therapy in response to physiological changes. The review then describes the sensing, detection, and stimulation technology that closes the loop and presents clinical experiences of the systems. | Not specified. | To focus on the clinical experience of four implantable closed-loop neurostimulation systems: positional-adaptive spinal cord stimulation for treatment of pain, responsive cortical stimulation for treatment of epilepsy, closed-loop vagus nerve stimulation for treatment of epilepsy, and concurrent sensing and stimulation for treatment of Parkinson disease. |  |
|  | Thanaviratananich et al  (abstract) | 2017 | USA | Research | Prospective study whereby home video recordings were analysed by a blinded experienced observer at the epilepsy clinic and a diagnostic impression was formed. | Adults 18 years and over. Video recordings were taken in the home. | To assess how well home video distinguishes epileptic seizures from other paroxysmal events (other spasms or seizures) by assessing its sensitivity, specificity, positive predictive value (PPV)/negative predictive value (NPV). |  |
|  | van Andel et al | 2017 | The Netherlands | Research | Algorithm performance testing in a multi-centre prospective cohort study. | Data collected from 95 inpatients (due to sensor errors data included from 43 patients only, age range 2-65). | To develop an algorithm that can detect a broader range of seizure types, including tonic, hypermotor, and clusters of seizures using non-EEG signals (heart rate and movement). |  |
|  | van Bussel et al  (abstract) | 2012 | The Netherlands | Research | Observational study for the purpose of algorithm development. | 13 patients in an inpatient hospital setting. Data was reported on 3 patients. | To develop and validate a wearable cardiac monitor for real-time detection of major epileptic seizures. |  |
|  | Van de Vel et al | 2016b | Belgium | Research | Observational study | Video, accelerometry (ACM), and radar-induced activity recording during sleep. Two patients with tonic–clonic and clonic seizures, measured for about one month in a home environment with four wireless accelerometers (ACM) attached to wrists and ankles. Patient 1 – 8 year old girl with epilepsy. Patient 2- 9 year old boy with epilepsy | To test the efficacy of the VARIA system (video, accelerometry, and radar-induced activity recording) and validation of accelerometry-based detection algorithms for nocturnal tonic–clonic and clonic seizures developed by our team. |  |
|  | Van de Vel et al | 2016a | Belgium | Review | Descriptive review | Not specified | To update a previous literature review of non-invasive, non- electro-encephalography body signals that can be measured, along with corresponding methods, state of the art research, and commercially available systems. |  |
|  | Van de Vel et al | 2013 | Belgium | Review | Descriptive review | Not specified | This paper reviews non-electroencephalography seizure prediction systems to prevent SUDEP. It aims to provide healthcare professionals, engineers and interested patients with an overview of body signals that can be measured along with the corresponding methods of measurement, state-of-art research and available systems. |  |
|  | Velez et al | 2016 | USA | Research | Prospective feasibility study | 30 adult patients admitted for usual clinical purposes in an in epilepsy monitoring unit. To provoke seizures anti-seizure medications were tapered, patients were sleep deprived and performed stationary bike exercises | The test the feasibility of detecting and logging generalised tonic-clonic seizures with a wrist accelerometer and linking the data to an online seizure database. |  |
|  | Walker et al | 2012 | USA | Research | Secondary content analysis of data collected in the pilot and efficacy studies of the WebEase online self-management intervention. | 35 participants from the pilot (aged 20-63) and 148 from the main WebEase studies (aged 18-77). | To understand who people with epilepsy identify as supportive, and how those individuals support self-management efforts. |  |
|  | Walker et al | 2009 | USA | Research | Secondary content analysis of self-management goals entered on to the WebEase platform by people living with epilepsy. | 35 participants (aged 20-63) | To describe the content and characteristics of self-management goals set by individuals with epilepsy participating in the online self-management WebEase program. |  |
|  | Zhang et al  (abstract) | 2017 | USA | Research | Prospective study using mixed-methods to capture response rates, percentage of patents who reported seizure activity, message fatigue and the number of seizures affected by the system; as well as interviews to capture the subjective experience of using EpxEpilepsy. | Nine patients studied in a community setting. | To present pilot results from the implementation of EpxEpilepsy, an automated service that uses text messages and phone communications to support patients in monitoring seizure events and in communicating with their service providers. |  |

Table 2: Study characteristics of 59 articles resulting from original search 2 (on living with epilepsy) and subsequent citation tracking

|  | **Name** | **Year** | **Country** | **Type of paper** | **Study design/ methods** | **Sample/setting** | **Objectives** |
| --- | --- | --- | --- | --- | --- | --- | --- |
|  | Admi and Shaham | 2007 | Israel | Research | Qualitative life history method | 14 adolescents and young adults with epilepsy | To determine the centrality of epilepsy in the lives of the participants, to examine their view of others’ perceptions of people with epilepsy, to analyze their ways of managing disease-related information, and to analyze their ways of managing medical regimens. |
|  | Baker et al | 2001 | UK | Research | Quantitative cross-sectional study drawing on existing dataset from the records of 31 general practitioners | 669 older adults (cut-off point of 60 years in women and 65 years in men) with diagnosed epilepsy | To compare and contrast the Quality Of Life (QOL) profile of older vs. younger people with epilepsy, and whether age of onset of epilepsy is a critical variable determining QOL. |
|  | Borghs et al | 2016 | USA | Research | Conceptual literature review, followed by qualitative expert and patient interviews. | Four experts and 25 epilepsy patients (18-70 years old) were interviewed at four sites in the USA. | To understand patient experiences of seizure severity and to build a conceptual model. |
|  | Chung et al | 2012 | USA | Research | Qualitative focus groups and QOLIE-31-P survey instrument. | Six focus groups were conducted in the San Francisco Bay area. | To evaluate quality of life for people living with epilepsy. |
|  | Collard and Ellis-Hill | 2017a | UK | Research | Qualitative (focus groups and semi-structured interviews; drawing on constructionist grounded theory) | Three focus groups and 3 online interviews conducted with 11 participants; people over 18 with epilepsy (mean 42 years) and no other co-morbidities that would prevent exercise. | To explore ways of enhancing physical activity levels for people living with epilepsy by examining the barriers to exercise, current adaptation techniques used to overcome these barriers, and common exercise activities and intensity levels. |
|  | Collard and Ellis-Hill | 2017b | UK | Research | Qualitative (focus groups and semi-structured interviews; drawing on constructionist grounded theory) | Three focus groups and 3 online interviews conducted with 11 participants; people over 18 with epilepsy (mean 42 years) and no other co-morbidities that would prevent exercise. | To explore the impact of stigma for people with epilepsy in terms of their engagement with exercise and identifying strategies for reducing negative effects. |
|  | Collard and Marlow | 2016b | UK | Research | Qualitative (interviews drawing on a social constructionist philosophy and narrative inquiry) | Total 16 semi-structured/conversational interviews; 4 with each participant. Total 4 participants with epilepsy as their main condition, between 23-38 years old. | To develop a better understanding of the psychosocial impact of exercising with epilepsy. |
|  | Collard and Marlow | 2016a | UK | Research | Qualitative interviews (interviews drawing on a social constructionist philosophy and narrative inquiry) | Total 16 semi-structured/conversational interviews; 4 with each participant. Total 4 participants with epilepsy as their main condition, between 23-38 years old. | To present the psychosocial impact of exercising with epilepsy over time and encourage the development of methods for people with epilepsy to overcome barriers and benefit from physical activity. |
|  | Dennis | 2013 | USA | Research (dissertation) | Qualitative (interviews, online content analysis and pre-/post- acceptability evaluation) | One year of reproductive-focused postings to online forums for women with epilepsy and 30 in-depth interviews with women with epilepsy of reproductive age. Development of a contraceptive decision aid and pre-post evaluation with 14 women with epilepsy. | To explore reproductive decision-making processes for women with epilepsy, and their experiences and needs for informed decision-making, and to develop an intervention tool responsive to these needs. |
|  | Fernandes et al | 2011 | UK | Discussion paper | Conceptual discussion and literature review | N/A – studies included from all over the world but focussed predominantly on OECD contexts | To discuss the concept of stigma (felt and enacted) through psychosocial theories and to present promising areas of research. |
|  | Gauffin et al | 2011 | Sweden | Research | Qualitative focus group study | Four focus groups with 14 adults (18-35 years old) diagnosed with epilepsy for at least 1 year; using AEDs and with subjective memory problems. | To explore the experience of living with epilepsy and subjective cognitive difficulties |
|  | Harden et al | 2015 | UK | Research | Qualitative interviews | 27 young adults (aged 18–29 years) with epilepsy | To explore the views and experiences of young adults with epilepsy on the risks associated with, and information giving in relation to sudden unexpected death in epilepsy (SUDEP) |
|  | Herrmann et al | 2016 | US | Review | Systematic review of literature on epilepsy stigma published 2004-2015. | The review included studies from Europe, the Americas, and Australia, on studies of about perceptions of epilepsy among people who did not have epilepsy in the general population (rather than self-perceived stigma) | To understand the recent literature on misconceptions and stigma surrounding epilepsy in Western societies and to identify key information relevant to understanding and modifying these misconceptions in order to limit stigma in Western societies. |
|  | Jacoby | 1994 | UK | Research | Quantitative survey study using a combination of pre-coded and open questions, including validated scales. | 607 epilepsy patients identified through a multicentre randomised controlled trial. One of the eligibility criteria was freedom from seizures for minimum two years. | To describe the nature and extent of stigma in a group of individuals with epilepsy in remission. |
|  | Jacoby et al | 2005 | UK | Review | Narrative review using a structured search strategy and set exclusion criteria, across 9 databases and grey literature sources. | n/a | To present general theories of stigma and relate these to the stigma associated with epilepsy throughout history and across cultures. |
|  | Jacoby et al | 2014 | UK | Research | Qualitative interviews | 67 adult participants recruited from UK-based RCT on AEDs. | To explore the utility of the concept of loss and loss replacement as a means of gaining a fuller understanding of the implications of a diagnosis of epilepsy for overall quality of life. |
|  | Kerr et al | 2011 | UK | Systematic review | Systematic search in Embase and Medline, followed by extraction of qualitative data on the impact of epilepsy on children and on the impact of epilepsy on adults. | N/A | To develop a conceptual model of the impact of partial onset or generalized epilepsy on children and adults in order to guide the identification of endpoints that capture patient perspectives in new treatment trials. |
|  | Kilinc and Campbell | 2009 | UK | Research | Qualitative (semi-structured interviews using a phenomenological approach). | 52 interviewees (19-57 years old) diagnosed with epilepsy after the age of 18. Of those, 32 also participated in follow-up interviews up to 1 year later. | To explore the experience of stigma for adults with epilepsy. |
|  | Kilinc et al | 2017 | UK | Research | Qualitative (semi-structured interviews using a phenomenological approach). | Thirty-nine participants were recruited in total (14 men and  25 women). They were diagnosed with epilepsy between 18 and 57 years of age. Of those, 24 took part in a  follow-up interview (6 men and 18 women). | To produce an in-depth analysis of what it means to live with adult onset epilepsy from an experiential perspective. |
|  | Leppik | 2006 | USA | Review | Descriptive synthesis of clinical evidence | n/a | To review the literature on clinical use of anti-epileptic drug treatment in older people. |
|  | Lua and Neni | 2013 | Malaysia | Research (interventional) | Randomized, controlled open-label trial: the control group received an eleven-part printed epilepsy educational module to be completed over 12 weeks and the intervention group in addition received an SMS-based intervention.  Statistical methods were used to compare control and intervention groups. | Total of 144 people living with epilepsy (mean age 30.5 ± 11.8 years) from outpatient clinics in three public hospitals in Malaysia. | To investigate the impact of SMS-based telemedicine education on people living with epilepsy and their health-related quality of life status. |
|  | Lyons and Dolezal | 2017 | Ireland | Commentary | n/a | n/a | To introduce a special issue on shame and stigma in medicine, including on infectious disease, the dynamics of the clinical encounter, chronic pain, and subjective experiences in mental health. |
|  | Mahrer-Imhof et al | 2013 | Switzerland | Research | Cross-sectional exploratory study using statistical methods | One hundred and four dyads of adult patients and their main care-giving family members took part. Patients were hospitalized in a Swiss tertiary epilepsy clinic. | To investigate factors that influence quality of life in hospitalized adult patients with epilepsy and their relatives. |
|  | Martin et al | 2003 | USA | Review | Update of previous systematic review, following a search in 2 databases and hand-searching between 1998-2002. | Studies were reviewed if they included a randomised controlled trial design and included epilepsy patients over the age of 60. | To assess current state of knowledge pertaining to antiepileptic drug treatment effects on health-related quality of life in seniors with epilepsy. |
|  | Martin et al | 2005 | USA | Research | Survey-based methods using an unstructured approach to eliciting concerns, which were then elaborated in a small number of interviews and tabulated for the analysis. | 33 participants with experience of seizures living independently in the community and at least 60 years old | To examine the concerns of living with recurrent seizures as expressed by older adults |
|  | McLaughlin et al | 2008 | Australia | Research | Quantitative study using instruments such as the mini mental state exam (MMSE), the Washington Psychosocial Seizure Inventory (WPSI), and Quality of life in epilepsy (QOLIE-31), among others. | 64 community dwelling older adults (over 60) with a confirmed diagnosis of epilepsy | To examine the impact of perceived stigma and seizure frequency on health-related quality of life and psychosocial wellbeing in late adulthood. |
|  | Mengoni et al | 2016 | UK | Research | Qualitative interviews | Fifteen people with intellectual disabilities and epilepsy and their carers. | To investigate the impact and management of epilepsy in people with intellectual disabilities. |
|  | Miller et al | 2014 | USA | Research | Qualitative interviews | Convenience sample of 20 adults (mean age 70) diagnosed with epilepsy at 60 or over. | To determine perceived self-management problems and needs for older people with epilepsy, as well as strategies used to address problems and needs. |
|  | Monzoni and Reuber | 2009 | UK | Research | Interactional and linguistic analysis of communication behavior in first interaction between patients and their neurologist; using discourse psychology and conversation analysis. | Transcripts of video recordings of 30-minute clinical encounters between nine patients with epilepsy and a neurologist. | To explores whether close examination of the interactional and linguistic resources used by PWE can be used to enhance our understanding of coping behaviour. |
|  | Pembroke et al | 2017 | Ireland | Research | Qualitative interviews and grounded theory | 49 adults with a diagnosis of epilepsy who expressed feeling comfortable with their epilepsy. | To explore how people with epilepsy become comfortable with their epilepsy and contribute to effective self-management strategies for those newly diagnosed. |
|  | Perzynski et al | 2017 | USA | Research | Qualitative focus group study using thematic, constant comparative analysis. | Six focus groups as part of community advisory board meetings. There were a total of 22 participants, including 8 health professionals, 9 patients with epilepsy, and 5 care partners. | To identify barriers and facilitators in epilepsy self-management for those with other physical or psychological conditions. |
|  | Raty and Wilde-Larsson | 2011 | Sweden | Research | Qualitative interviews using a phenomenographic approach. | 19 outpatients with epilepsy recruited from a neurology clinic. | To describe how patients perceive living with epilepsy. |
|  | Raty et al | 2007 | Sweden | Research | Questionnaire including open-ended questions, analysed using content analysis and the Belief Desire Theory of Emotions. | 95 young adults (18–27 years). | To illuminate the impact of epilepsy on the daily life of young adults as described through their emotions. |
|  | Raty et al | 2009 | Sweden | Research | Qualitative interviews using a phenomenographic approach. | 19 outpatients with epilepsy recruited from a neurology clinic. | To highlight epilepsy patients’ conceptions of epilepsy as a phenomenon and emotions related to those conceptions. |
|  | Rawlings et al | 2017 | UK | Research | Qualitative textual analysis drawing on written pieces produced by patients for the study. | 20 participants (17 female) recruited from a hospital and membership-led organizations. | To examine the subjective experience of living with epilepsy by thematically analysing participants' written accounts of their condition. |
|  | Reis and Meinardi | 2002 | Netherlands | Commentary/discussion paper | n/a | n/a | To discuss stigma in epilepsy, including its definition, differences across cultures, and possible steps to help eliminate it. |
|  | Rhodes et al | 2008 | UK | Research | Qualitative interviews and focus groups. | Adults of Pakistani Muslim origin resident in Bradford, with epilepsy and no learning disability.  Participants also nominated a carer to be interviewed. | To explore attitudes towards their condition; their perceptions of other people’s attitudes; impact on their lives; conceptions of disability; and self-identification as disabled. |
|  | Ring et al | 2016 | UK | Research | Qualitative interviews | 67 adults with epilepsy (24-65 years old) | To understand the factors that influence quality of life for people living with epilepsy and present a model of the complex influences mitigate or further compound the negative impacts of epilepsy. |
|  | Ryan and Raisanen | 2012 | UK | Research | Qualitative in-depth interviews | 37 young people living with epilepsy (aged 16-28) recruited through epilepsy support groups and organisations, internet forums, schools and colleges, GPs, neurologists and specialist nurses. | To explore the emotional experience of young people with epilepsy. |
|  | Scambler | 1989 | UK | Book | Multiple | Multiple | This book presents a collection of studies on epilepsy focusing on the social aspects of living with the condition: experiences of diagnosis, stigma, coping strategies, impact on family life, and work and inequalities. |
|  | Scambler | 2004 | UK | Theoretical development | n/a | n/a | To revisit the hidden distress model of epilepsy in light of contemporary theory and present a new research agenda for sociologists interested in epilepsy. |
|  | Shi et al | 2017 | China | Systematic review | Systematic searches in 4 databases followed by meta-analysis. | Primary research papers evaluating sociodemographic, psychosocial, and disease-related variables purported to have a statistical relationship with perceived stigma for people living with epilepsy. | To identify the correlates of perceived stigma for people living with epilepsy. |
|  | Shostak and Fox | 2012 | USA | Research | Secondary analysis of qualitative interviews | 80 interviews with people living with epilepsy (and family members). One set of interviews collected in 1975-1978 and another in 2005-2006. | This article examines how memories of what epilepsy has been shape the individual and collective identities of people living with epilepsy. |
|  | Sleeth et al | 2016 | USA | Research | Qualitative interviews | 57 individuals aged 65 years or older (mean 74) who had seizures or had been diagnosed with epilepsy (majority in their 60s). | The aim of this study was to qualitatively assess the effects of stigma on the quality of life of older people with epilepsy. |
|  | Stepney et al | 2018 | UK | Research | Secondary analysis of qualitative interviews - focusing on narratives of driving. | 154 interview transcripts on people’s experiences with motor neurone disease, epilepsy, Parkinson’s and minor stroke. | To understand the significance of driving for people with neurological conditions and the impact of the withdrawal of a driving licence, taking an interactionist perspective. |
|  | Tebartz et al | 2009 | Germany | Commentary | n/a | n/a | To review and summarize the clinical and psychosocial aspects of epilepsy for older-age patients. |
|  | Thompson et al | 2008 | UK | Research | Qualitative interviews | 15 women aged 20-40 with epilepsy; most had been diagnosed in childhood or adolescence. Ten women had at least one child. | To explore the reproductive experiences of women with epilepsy. |
|  | Thompson et al | 2013 | UK, Ireland | Research | Survey questionnaire (online) | 113 health professionals and caregivers, both paid and family members. | To examine the impact of epilepsy on individuals with intellectual disability and on their caregivers and to elicit views on treatment available for this population. |
|  | Unger and Buelow | 2009 | USA | Research | Hybrid concept analysis method, consisting of a conceptual phase (literature review and content analysis) and an empirical phase (qualitative interviews) | Four adults were recruited through the neurology clinic. | To analyse and define the concept of self-management for adults diagnosed with epilepsy in the previous 12 months. |
|  | Vadrot | 2013 | Austria | Research | Ethnographic study and review of scientific papers | Interviews conducted with patients, medical doctors/researchers, and technicians involved in a Video-EEG-Monitoring project (VEM) in a neurological centre. | To better understand how the convergence of technologies in the field of neuroscience takes place in the clinic. |
|  | Walker et al | 2014b | USA | Research | Sequential mixed methods study, including a telephone survey and in-depth interviews. | 101 individuals completed the survey and 38 participated in interviews (including people living with epilepsy and their support persons). | To examine patterns of epilepsy self-management support from the perspectives of people living with epilepsy and their support persons |
|  | Walker et al | 2014a | USA | Research | Qualitative interviews using grounded theory | In-depth interviews with 38 individuals (22 people living with epilepsy and 16 support persons). | To examine patterns of epilepsy self-management support from the perspectives of people living with epilepsy and their support persons |
|  | Walker et al | 2015 | USA | Research | Qualitative interviews using grounded theory | In-depth interviews with 38 individuals (22 people living with epilepsy and 16 support persons). | To examine the experiences of people with epilepsy and their support persons and to explore interpersonal relationships between dyad members. |
|  | Weckesser and Denny | 2013 | UK | Systematic review | Systematic literature search in medical and social science databases to identify qualitative research and questionnaires with open ended questions. | The review included qualitative studies on reproduction and epilepsy; questionnaire studies using open-ended questions; qualitative studies on pregnancy and reproductive issues. | To investigate the experiences of pregnancy in women living with epilepsy from preconception to post- delivery. |
|  | Weckesser and Denny | 2017 | UK | Research | Qualitative interviews | 32 pregnant women with epilepsy participated in interviews; 24 also took part on follow-up post-natal interviews. | To explore how pregnant women living with epilepsy re-work their biographies in the context of the moral work of managing epilepsy. |
|  | Wedlund et al | 2013 | Sweden | Research | Qualitative focus groups | Six focus groups; five with patients following comprehensive rehabilitation in a day case setting (n=17) and one with staff members (n=5). | To identify issues experienced as important in the rehabilitation for persons with epilepsy. |
|  | Widnes et al | 2012 | Norway | Research | Interviews with pregnant women with epilepsy treated with AEDS. | Ten women 22-39 years old in 20-34 weeks’ gestation. | To explore risk perception and medicines information needs in pregnant women with epilepsy. |
|  | Yennadiou and Wolverson | 2017 | UK | Research | Qualitative interviews based on the common-sense model of illness representations (CSMIR) and using Interpretative Phenomenological Analysis. | Ten patients with a confirmed diagnosis of epilepsy who were over 65 years old. | To explore the lived experience of epilepsy in later life through older peoples' appraisals of their condition. |
|  | Yuen et al | 2018 | UK | Review | Conceptual discussion. | n/a | To present an approach to epilepsy, not just as a neurological condition, but as a systemic dysfunction. |

Table 3: Study characteristics of 23 articles resulting from updated search 1 (technology and epilepsy)

|  | **Name** | **Year** | **Country** | **Type of paper** | **Study design/method** | **Sample/setting** | **Objective** |
| --- | --- | --- | --- | --- | --- | --- | --- |
|  | Afra et al | 2018 | USA | Research | Survey-based study and design of prototype mobile software to reduce epileptic seizures | Total 40 patients living with epilepsy completed the survey. The study was carried out at University of Utah Adult Comprehensive Epilepsy Clinic and recruited patients aged 18 years and older, with no cognitive impairments, who were regular smartphone users. | To examine patient preferences in relation to proposed features of a mobile app used as ‘digital therapeutic’ for seizure control and to present the design of a proof-of-concept mobile software providing digital content as non-pharmacological intervention. |
|  | Amengual-Gual et al | 2018 | USA | Clinical review | Descriptive | n/a | To summarize the evidence demonstrating a non-random pattern of seizure occurrence, the potential mechanisms that explain these cyclical patterns or rhythmicity, and the implications for seizure prediction and seizure treatment. |
|  | Bates | 2018 | USA | Article in scientific magazine | Journalistic description | n/a | To provide an overview of recent developments in technologies that support monitoring, responsive neurostimulation and prediction of seizures in epilepsy (e.g. NeuroPace, EpiWatch). |
|  | Baumgartner et al | 2018 | Austria | Review | Descriptive | n/a | To review automatic seizure detection based on scalp electroencephalography (scalp-EEG), electrocardiography (ECG) and surface electromyography (sEMG). |
|  | Beniczky et al | 2018a | Denmark and Norway | Research | Prospective, multicenter study. Performance of an EMG-based wearable device was compared to the evaluation of video-EGG recordings by trained experts who were blinded to data from the device. | 71 patients (32 female, age 19-61 years, mean 34.1, median 34) recruited in three epilepsy monitoring units. | To test the technical performance and diagnostic accuracy of real time seizure detection using a wearable surface EMG device. |
|  | Beniczky et al | 2018b | Denmark | Review | Descriptive | n/a | This article summarizes the pathophysiology of muscle activation during convulsive seizures and reviews the published evidence on the accuracy of EMG-based seizure detection |
|  | Bruno et al | 2018 | UK | Research | Online survey | Total 87 participants, including people with epilepsy, caregivers and health professionals. | To explore willingness to use wearables to monitor seizures and factors influencing engagement with technology. |
|  | Carmenate et al  (abstract) | 2017 | USA | Research | National, prospective study, drawing on data analysis of Epiwatch that uses Apple Watch to track heart rate, movements, and responsiveness during seizures. This was followed by a post-seizure survey | 704 participants (mean age: 31, range: 16-73), with  3026 seizures recorded | To report an initial analysis of heart rate changes associated with seizures. |
|  | Casassa et al | 2018 | USA | Review/ commentary | Descriptive. | Epilepsy monitoring applications | Provides an overview of a small number of epilepsy-related technological applications (seizure tracker, my seizure diary, EpiDiary), discussing their benefits and limitations. |
|  | Dumanis et al | 2017 | USA | Review/  commentary | Drawing on themes identified in an online survey and innovation workshop. | n/a | To introduce the work of the Epileptic Institute (EI) and their focus on prioritisation of patient needs, including an individualized seizure advisory device that will allow a person with epilepsy to monitor the likelihood of a seizure on a daily basis. |
|  | Escoffery et al | 2018 | USA | Systematic review | Systematic search on iTunes app store and screening based on inclusion/exclusion criteria, followed by structured data extraction. | Apps focused on epilepsy that were developed for people living with epilepsy or for the public; 149 apps were identified of which 20 met selection criteria. | To review mobile applications related to epilepsy self-management. |
|  | Geertsema et al | 2018 | Netherlands | Research | Computational algorithm training and testing. | Residential care setting. The test set consisted of data from 12 patients spending 24 full nights in residential care and additional recordings of 50 seizures selected randomly to estimate performance; data were analyzed retrospectively. | To investigate the detection performance of a video algorithm to detect convulsive seizures |
|  | Johansson et al | 2018 | Sweden | Systematic review | Search strategies were structured based on the PICO framework and SPIDER tool. They were run on two databases. Screening and quality assessment preceded thematic analysis. | 56 qualitative and quantitative studies included. | To review knowledge from papers analysing wearable sensors in epilepsy, PD and stroke. |
|  | Kurada et al | 2019 | USA | Systematic Review | Following PRISMA criteria; the search was run on PubMed only and screening followed specific criteria. | 36 device validation studies included | To characterise current capabilities of seizure detector devices and to assess their appropriateness for use in clinical trials. |
|  | Kusmakar et al | 2018 | Australia | Research | Validation study using data from a single wrist-worn accelerometer device for ambulatory monitoring for detection of convulsive epileptic seizures. | A total of 79 patients were recruited between 2012 to 2015. | To present the performance evaluation of a novel seizure detection algorithm sensitive to different seizure types, even those with short duration. |
|  | McLean et al | 2017 | UK | Review | Descriptive | n/a | Assess strategies and technological solutions in reducing risk of SUDEP. |
|  | Meritam et al | 2018 | Denmark | Research | Field study using a usability questionnaire. | 71 patients in community who used a wearable accelerometer device (Epi-Care) | To demonstrate applicability and usability of an accelerometer device for detecting bilateral tonic clonic seizures |
|  | Olsen et al  (abstract) | 2017 | Denmark | Clinical improvement | Description of technology- supported clinical service. | 600 users reported | Introducing the Seizure App used by patients in the Danish Epilepsy Centre |
|  | Page et al  (abstract) | 2018b | UK | Research | One year pilot including measures such as hospital admissions, seizure notification, and other clinically relevant outcomes. | Patients in the community used a smart phone and wearable technology to notify clinicians of seizures and clinically relevant information. | To demonstrate that technological approaches can be used successfully to improve care of epilepsy patients. |
|  | Page et al  (abstract) | 2018a | UK | Research | One year pilot (same as above). This abstract also reports additional measures such as emergency attendance and length of stay, and empowerment measures. | 55 patients with a diagnosis of epilepsy were recruited from a specialist epilepsy clinic. | To evaluate the potential of a novel technological solution for supporting patients with epilepsy as part of specialist epilepsy care. |
|  | Todd et al  (abstract) | 2017 | Australia | Research | Consumer and stakeholder consultation, analysis of support service requests and review of quality of life and self-management literature. | 62 service users piloted and evaluated the technology. | To test the feasibility of QR code/USB key accessible microsite (myepilesykey) with evidence based epilepsy content. |
|  | Yasam et al | 2018 | India | Review | Descriptive. | n/a | Overview of non-drug therapies, including monitoring devices, for treatment and management of epilepsy. |
|  | Zhao and Lhatoo | 2018 | USA | Review | Descriptive | n/a | To summarise available EEG-based and non-EEG-based detection systems for epileptic seizures in the ambulatory setting. |

Table 4: Study characteristics of 13 articles resulting from updated search 2 (on living with epilepsy)

|  | **Name** | **Year** | **Country** | **Type of paper** | **Study design/ methods** | **Sample/setting** | **Objectives** |
| --- | --- | --- | --- | --- | --- | --- | --- |
|  | Celani et al | 2018 | Italy | Research | Focus groups with participants and carers divided into severity levels of disease; data analysed quantitatively. | 25 patients and 36 carers attending outpatient services in a tertiary epilepsy centre. | To elicit perspectives, ideas, and values from people affected by epilepsy and their carers to help shape the epilepsy service. |
|  | Fitzsimons et al  (abstract) | 2017 | Ireland | Research/clinical improvement | Participatory action research including in-depth interviews, ethnographic observations and focus groups. | 32 people with epilepsy, 6 community resource officers, 4 consultant epileptologists, 13 epilepsy specialist nurses, 3 intellectual disability sector nurses, 3 general practitioners, 2 health service managers and 1 epilepsy service manager at various sites across Ireland. | To co-design integrated care for people living with epilepsy in Ireland. |
|  | Kilinc et al | 2018 | UK | Research | Semi-structured interviews followed by a modified form of interpretative phenomenological analysis to identify themes. | 39 people with adult onset epilepsy from across the UK | To explore what the experience of adult-onset epilepsy meant for a person’s identity. |
|  | Lapalme-Remis et al  (abstract) | 2017 | USA | Research | Qualitative content analysis. | Online epilepsy support group discussions. | To understand the issues most important to patients to promote patient-centred care in epilepsy. |
|  | Lawther et al | 2018 | UK | Research | Observation and semi-structured interviews, followed by interpretative phenomenological analysis. | Purposive sample: 7 women from preconception clinic and from antenatal clinic in one region of the UK. All had taken valproate preconceptually. | To understand the preconception experiences of women with epilepsy who have been taking the teratogenic drug valproate. |
|  | Moccia et al | 2018 | Italy | Descriptive review | Not specified. | Not specified. | To discuss how the internet is affecting the healthcare of people with neurological disorders and shifting the paradigm of care from the hands of those who deliver care, into the hands of those receive it- and to review possible limitations, such as safety concerns, financial issues and the need for easy to access platforms. |
|  | Ninnoni | 2019 | UK | Research | Descriptive qualitative approach using semi-structured interviews. | 15 people with mild learning disabilities and epilepsy, based in the community, and 13 carers in the north of Scotland, purposively sampled and recruited through learning disabilities teams. | To investigate the communication and information needs of people with learning disabilities and epilepsy. |
|  | Power et al  (abstract) | 2017 | Ireland | Research/service improvement | A socio-technical process based on ethnographic analysis, interviews, focus groups, and joint design workshops was conducted to establish stakeholder needs and inform the design of the patient portal. | Not specified | To design, develop, implement and evaluate a patient portal to the National Epilepsy Electronic Patient Record (EPR) in Ireland. |
|  | Rawlings et al | 2018a | UK | Research | Participants were given writing booklets and asked to complete 4 pieces of writing centered around pre-specified topics. Qualitative thematic analysis was undertaken on the written pieces. | 19 participants with psychogenic non-epileptic seizures and 20 with diagnosed epilepsy, over 18 years old, recruited from outpatient neurology clinics in Sheffield, UK. | To examine the subjective experience of living with epilepsy or psychogenic non-epileptic seizures (PNES) by thematically comparing individuals’ written accounts of their condition |
|  | Rawlings et al | 2018b | UK | Research | As part of a therapeutic writing intervention for individuals living with a seizure disorder, participants were asked to write for at least 20 min about their very deepest thoughts and feelings about their condition. Qualitative narrative analysis was undertaken. | Patients with epilepsy (n = 29) or psychogenic non-epileptic seizures (n = 20). | To use narrative analysis to identify common storylines, otherwise known as narrative typologies of individuals’ written accounts of living with epilepsy |
|  | Sallay et al | 2019 | Hungary | Research | In-depth semi-structured interviews. Authors analysed the data through grounded theory. | 23 adults from 10 families: four families with a person affected by epilepsy, six families with a person affected by chronic back pain. The sample included 13 women and 10 men, aged between 25 and 57. | To highlight the complex coping processes in the family home, and self-regulation processes in the context of chronic illness by analysing participants’ emotionally significant experiences. |
|  | Scott et al | 2018 | Australia | Research | Mixed-methods using self-reported demographic and epilepsy questionnaires and semi-structured interviews. Grounded theory analysis was used to develop a theoretical model of anxiety development in the context of epilepsy. | 26 adults with epilepsy, recruited from an outpatient epilepsy service. | To determine what factors may explain why some people with epilepsy develop anxiety disorders and others do not. |
|  | Varley et al  (abstract) | 2017 | Ireland | Research | Co-design approach drawing on ethnography, interviews and focus groups in parallel with participatory action research (PAR) | 32 people with epilepsy, 6 community resource officers, 4 consultant epileptologists, 13 epilepsy specialist nurses, 3 intellectual disability sector nurses, 3 general practitioners, 2 health service managers and 1 epilepsy service manager have participated in the project | To develop a participatory approach to the design and development of epilepsy health services. |

Table 5: Indicative list of studies per category

| **Categories** | **Illustrative technology studies** |
| --- | --- |
| **Computational view**  Having the potential to be optimised through programming for maximum effect, especially in terms of computational power and capability. | Presentation of technical seizure detection system components and architecture (Bonnet et al., 2011)  Computational platform developed for training and testing seizure prediction algorithms, establishment of the European Epilepsy Database of EEG recordings and the Brainatic hardware for seizure prediction, following testing of different technical configurations (Dourado et al., 2009; Dourado et al., 2014)  Generalized tonic-clonic seizure detection using modified algorithms to reduce computational load, followed by data training and initial small-scale testing in epilepsy monitoring units (Conradsen et al., 2012)  Algorithm implementation and evaluation (Borujeny et al., 2013)  Building patient-specific models from accelerometer data, modelling normal movement and then detecting abnormal events to distinguish between epileptic and non-epileptic movements, rather than relying on database training data (Cuppens et al., 2014)  Algorithm development for epileptic seizures with and without motor activity (Heldberg et al., 2015)  Multi-biosignal analysis for epileptic seizure monitoring (Cogan et al., 2017)  System implementation and testing methodology for a tablet-based seizure monitoring and classification system (Gouravajhala et al., 2012)  Biosignal processing and interpretation in telemonitoring for epilepsy patients (Jones et al., 2008)  Ambulatory seizure monitoring (Myers et al., 2016)  Automatic prediction of epileptic seizures using cloud computing and wireless sensor networks (Sareen et al., 2016)  Smart mobile end-to-end monitoring (Serhani et al., 2016) |
| **Tool view**  Directly and ‘objectively’ capturing data on the condition, enhances monitoring accuracy and allows better data aggregation and information processing based on pre-defined measures. | Seizure detection sensors and systems (Beniczky et al., 2013; Bidwell et al., 2015; Carlson et al., 2009; Cuppens et al., 2014; Helmy et al., 2015; Jory et al., 2016; Kramer et al., 2011; Lockman et al., 2011; Nijsen et al., 2005; Patel et al., 2009; Patterson et al., 2015; Picard et al., 2017; Poh et al., 2012; Popescu et al., 2013; Poppel et al., 2013; Raam & Sasikala, 2016; Reeder & David, 2016; Regalia et al., 2015; Salem et al., 2014; Stigwall et al., 2011; van Andel et al., 2017; Van Bussel et al., 2012; Van de Vel et al., 2016a; Van de Vel et al., 2013; Van de Vel et al., 2016b; Velez et al., 2016)  Different physiological outputs used to detect seizures (with mixed results), including heart rate (Jones et al., 2008; Jory et al., 2016; van Andel et al., 2017), oxygen saturation, electrodermal activity (Heldberg et al., 2015; Poh et al., 2012; Ranganathan et al., 2015), muscle activity (Patel et al., 2009) or combinations (Cogan et al., 2017).  Physiological, clinical and technological issues and advances in seizure prediction (Gluckman & Schevon, 2015; Lehnertz, 2001; Litt & Echauz, 2002; Mormann et al., 2006; Nagaraj et al., 2015; Schwartz, 2007) including first-in-man studies of implantable devices (Cook et al., 2013; Elger & Mormann, 2013)  Distinguishing epileptic seizures from non-epileptic paroxysmal events, using home video (Thanaviratananich et al., 2017) and wearable devices (Gubbi et al., 2016)  Other applications, e.g. apnea detection to prevent SUDEP (Rodriguez-Villegas et al., 2009)  Online diary - Summary tables and charts can be printed and brought to clinic visits or e-mailed at time of clinic visits to allow copying and pasting directly into electronic medical records. (Fisher, 2010; Fisher et al., 2012)  Detecting and recording seizures accurately so that antiepileptic drugs can be adjusted accordingly (for a review see Bidwell et al., 2015)  Early detection of behavioral side effects of antiepileptic treatment using handheld computers (Frings et al., 2008)  Treatment delivery (Fisher, 2012; Litt, 2001; Morrell, 2009; Nagaraj et al., 2015; Stacey & Litt, 2008; Sun & Morrell, 2014)  Smartphone apps for medication tracking (Pandher & Bhullar, 2016; Ranganathan et al., 2015) |
| **Platform**  As conduit for education, peer support, self-monitoring and self-management. | Online program to provide education and support for self-management (DiIorio et al., 2011; DiIorio et al., 2009a)  Effectiveness of epilepsy-specific system (digital tools for tracking seizures, symptoms, medications, and comorbidities, along with access to an exclusive veteran discussion forum and specialised information provision) on the online PatientsLikeMe community (Hixson, 2016; Hixson et al., 2015a)  Self-management group intervention supported by eHealth tools (ZMILE): process evaluation of a group-based intervention programme in the Netherlands where, among its other aspects, patients were asked to use a compliance monitoring system (electronic caps fitted on pill bottles) and a smartphone application with a seizure diary, medication reminder function and alarm mode (Leenen et al., 2017)  Smartphone apps to fulfil educational needs, although a recent review found existing tools insufficient in how information was provided and presented (Pandher & Bhullar, 2016)  The smartphone app EpSMon (Epilepsy Self Monitor) allows users to carry out self-assessment of modifiable risk factors for SUDEP, aiming to educate patients and to encourage contact with a health professional where needed (Newman et al., 2016; Shankar et al., 2015)  ‘Managing epilepsy well’ network to advance epilepsy self-management and research by developing combination of interventions, including e-health strategies and decision support for patients (Sajatovic et al., 2017; Shegog & Begley, 2015) |
| **Intermediary**  Intermediary providing easier access to patients, making them knowable by mobilising specific types of data and ways of connecting, and often transcending physical distance between clinical teams, patients, researchers. | Remote consultations and video conferencing in epilepsy care (Patterson & Bingham, 2005)  Remote management of epilepsy and targeted communication with clinical team at the point of need (Page et al., 2016; Page et al., 2018c)  Technology-supported (including video-conferencing, wearable sensors) patient-centered population health management (PHM) outreach delivery model for chronic epilepsy care (Rossi et al., 2015)  Automated text messaging collecting seizure-related information from patients and transmitting this to providers, along with alerts for high-risk patients (Zhang et al., 2017)  Online and smartphone seizure diaries to record seizures, triggers, symptoms and other details that might aid patient self-management and contribute to decision-making by clinicians (Fisher, 2010; Fisher et al., 2015; Fisher et al., 2012; Pandher & Bhullar, 2016; Ranganathan et al., 2015). Examples include the ‘My Epilepsy Diary’, a web and mobile based service that allows patients to log seizure events, record medication, missed doses and side effects, set up alerts and graphically displays trends of seizure incidence (Fisher, 2010; Fisher et al., 2012). In a retrospective study involving 28,000 community-based patients using the ‘My Epilepsy Diary’, Fisher et al analysed self-reported diary data to describe prevalence and frequency of cluster seizures, and associated factors (Fisher et al., 2015).  EpiWatch smartphone app used for responsiveness testing along with biosensor data collected on the Apple watch – abstract reports on patient characteristics, seizure frequency and triggers, and medication adherence (Ge et al., 2017)  Online patient communities as a means of collecting data on patient characteristics, symptoms and medication side effects, and quality of life (de la Loge et al., 2016)  Drawing on data from seizure diaries to better understand premonitory symptoms and potential for self-prediction in patients with refractory focal epilepsy or to attempt predictive modelling of seizures (Hall et al., 2009a; Hall et al., 2009b; Haut et al., 2012; Haut et al., 2013)  Online seizure diary aimed at supporting clinical care – used in this study to provide a snapshot of patient characteristics, symptoms and seizures (Le et al., 2011)  Mobile phone application (diary) for tracking seizures, medication and fertility data in a controlled study evaluating fertility in women living with epilepsy – WEPOD study (Ernst et al., 2016; French et al., 2016; Llewellyn et al., 2013; Pennell et al., 2012)  Personal digital assistants (PDAs) used by patients to record prodrome sensations and seizure events, to understand if the former can predict the latter (Maiwald et al., 2011)  RCT of stress reduction with smartphone monitoring of stress and mood variables (Privitera et al., 2015)  Using the WebEase online self-management program to analyse goal setting by people living with epilepsy and to collect information on their perceptions of social support (Walker et al., 2012; Walker et al., 2009) |
| **Attitudes to technology**  Artifact (or imagined object) towards which potential users have more or less fixed attitudes (which are assumed to lead to either acceptance or rejection of the technology). | Survey on patient and caregiver perspectives around seizure prediction and acceptability of implantable devices (non-users) identified a significant need for new solutions to seizure management, but also a certain level of uncertainty and opposition towards device implantation (Arthurs et al., 2010)  Usability, feasibility and acceptability assessment of an online self-management programme (DiIorio et al., 2009a)  Patient attitudes towards seizure counting and assessment of the potential for electronic devices to facilitate tracking (Blachut et al., 2015)  Physician satisfaction with teleneurology service (Harper et al., 2016)  Satisfaction with online digital health management platform (Hixson et al., 2015a)  Acceptance of a mobile telecare system with tools for self-management and communication with clinical providers (Kabir et al., 2015)  Perceptions of usefulness and value of Dutch group-based multi-component self-management intervention for patients and their relatives; eHealth tools received low usefulness ratings, but technological potential still perceived important (Leenen et al., 2017)  Patient and caregiver attitudes towards and intention to use smartphone apps for epilepsy management (Liu et al., 2016a, 2016b)  Facilitators and barriers to using wearables for self-monitoring, including usability (Ozanne et al., 2017)  Patient and carer preferences in the design of wearable seizure detection devices (e.g. types of sensors, technical features), focused less on how the devices would fit in patients’ lives (Patel et al., 2016)  User retention in online digital health management platform (Hixson et al., 2015b)  Usage of e-Health tools by patients (generally assessed as low) (Leenen et al., 2016)  Rate of daily use and retention in the use of paper v electronic seizure diaries (Polak et al., 2014)  Uptake of EpSMon app (Newman et al., 2016) |
| **Dynamic socio-technical systems**  Actor or component in a wider network; a product of its conditions of development; enmeshed with the conditions of its use; implicated in sociocultural dynamics; embodying and recursively shaping social structures. | Detailed description of the development of a theory-based, online self-management programme (WebEase) and its different phases: literature review, survey on computer use and attitudes towards an internet-based programme, patient focus group, review by content experts and consumers, pilot study, along with lessons learnt from the development process (DiIorio et al., 2009b)  Development of EpSMon app (Newman et al., 2016)  Historical view on the development of engineered interventions for seizure prediction (Gluckman & Schevon, 2015)  Development of industry-wide standards and common data elements for mHealth in epilepsy (Goldenholz et al., 2018)  Extensive review of emerging technologies for the diagnosis, treatment and management of epilepsy, including assessment of potential for impact by health professionals and potential users (Horizon Scanning Research & Intelligence Centre, 2017)  How epilepsy as represented in socio-cultural imagination is heavily associated with metaphors of malfunctioning (digital) technologies and the black-boxed self (Roach, 2017) |

**References**

Admi, H., & Shaham, B. (2007). Living with epilepsy: ordinary people coping with extraordinary situations. *Qualitative Health Research, 17(9)*, 1178-1187.

Afra, P., Bruggers, C. S., Sweney, M., Fagatele, L., Alavi, F., Greenwald, M., . . . Bulaj, G. (2018). Mobile Software as a Medical Device (SaMD) for the Treatment of Epilepsy: Development of Digital Therapeutics Comprising Behavioral and Music-Based Interventions for Neurological Disorders. *Frontiers in Human Neuroscience, 12*, 171.

Amengual-Gual, M., Sanchez Fernandez, I., & Loddenkemper, T. (2019). Patterns of epileptic seizure occurrence. *Brain Research, 1703*, 3-12.

Arthurs, S., Zaveri, H. P., Frei, M. G., & Osorio, I. (2010). Patient and caregiver perspectives on seizure prediction. *Epilepsy & Behavior, 19(3)*, 474-477.

Baker, G. A., Jacoby, A., Buck, D., Brooks, J., Potts, P., & Chadwick, D. W. (2001). The quality of life of older people with epilepsy: findings from a UK community study. *Seizure, 10*(2), 92-99. <https://doi.org/https://doi.org/10.1053/seiz.2000.0465>

Bates, M. (2018). Controlling Seizures with Technology: Researchers Are Working to Predict and Prevent Epileptic Seizures Before They Happen. *IEEE Pulse, 9*(4), 25-28.

Baumgartner, C., Koren, J. P., & Rothmayer, M. (2018). Automatic Computer-Based Detection of Epileptic Seizures. *Frontiers in neurology, 9*. <https://doi.org/10.3389/fneur.2018.00639>

Beniczky, S., Conradsen, I., Henning, O., Fabricius, M., & Wolf, P. (2018a). Automated real-time detection of tonic-clonic seizures using a wearable EMG device. *Neurology, 90*(5), e428-e434.

Beniczky, S., Conradsen, I., & Wolf, P. (2018b). Detection of convulsive seizures using surface electromyography. *Epilepsia, 59 Suppl 1*, 23-29.

Beniczky, S., Polster, T., Kjaer, T. W., & Hjalgrim, H. (2013). Detection of generalized tonic-clonic seizures by a wireless wrist accelerometer: a prospective, multicenter study. *Epilepsia, 54(4)*, e58-61.

Bidwell, J., Khuwatsamrit, T., Askew, B., Ehrenberg, J. A., & Helmers, S. (2015). Seizure reporting technologies for epilepsy treatment: a review of clinical information needs and supporting technologies. *Seizure, 32*, 109-117.

Blachut, B., Hoppe, C., Surges, R., Elger, C. E., & Helmstaedter, C. (2015). Counting seizures: The primary outcome measure in epileptology from the patients’ perspective. *Epilepsia, 29*, 97-103.

Bonato, P. (2010). Wearable sensors and systems. From enabling technology to clinical applications. *IEEE Engineering in Medicine & Biology Magazine, 29(3)*, 25-36.

Bonnet, S., Jallon, P., Bourgerette, A., Antonakios, M., Guillemaud, R., Caritu, Y., . . . Ejnes, D. (2011). An Ethernet motion-sensor based alarm system for epilepsy monitoring. *Irbm, 32*(2), 155-157.

Borghs, S., Tomaszewski, E. L., Halling, K., & de la Loge, C. (2016). Understanding the Patient Perspective of Seizure Severity in Epilepsy: Development of a Conceptual Model. *The Patient: Patient-Centered Outcomes Research, 9(5)*, 419-431.

Borujeny, G. T., Yazdi, M., Keshavarz-Haddad, A., & Borujeny, A. R. (2013). Detection of epileptic seizure using wireless sensor networks. *Journal of Medical Signals & Sensors, 3(2)*, 63-68.

Bruno, E., Simblett, S., Lang, A., Biondi, A., Odoi, C., Schulze-Bonhage, A., . . . Consortium, R.-C. (2018). Wearable technology in epilepsy: The views of patients, caregivers, and healthcare professionals. *Epilepsy & Behavior, 85*, 141-149.

Carlson, C., Arnedo, V., Cahill, M., & Devinsky, O. (2009). Detecting nocturnal convulsions: efficacy of the MP5 monitor. *Seizure, 18*(3), 225-227.

Carmenate, Y. I., Gonzalez, E. M., Ge, A., Collard, M. J., Lee, S. W., Crone, N. E., & Krauss, G. L. (2017). Tracking heart rate changes during seizures: A national study using researchkit and apple watch to collect biosensor and response data during seizures. *Epilepsia, 58 (Supplement 5)*, S48.

Casassa, C., Rathbun Levit, E., & Goldenholz, D. M. (2018). Opinion and Special Articles: Self-management in epilepsy: Web-based seizure tracking applications. *Neurology, 91*(21), e2027-e2030.

Cavazos, J. E., Girouard, M., & Whitmire, L. (2015). Novel ambulatory EMG-based GTC seizure detection device for home and hospital use. *Journal of Clinical Neurophysiology, 32 (4)*, 394.

Celani, M. G., Cantisani, T. A., Bignamini, A., Macone, S., Papetti, R., & Baiocco, L. (2018). Is it worth it, or possible, to measure what matters to patients with epilepsy and their caregivers? *Epilepsy & Behavior, 78*, 273-279.

Chung, K., Liu, Y., Ivey, S. L., Huang, D., Chung, C., Guo, W., . . . Ma, D. (2012). Quality of life in epilepsy (QOLIE): insights about epilepsy and support groups from people with epilepsy (San Francisco Bay Area, USA). *Epilepsy & Behavior, 24(2)*, 256-263.

Cogan, D., Birjandtalab, J., Nourani, M., Harvey, J., & Nagaraddi, V. (2017). Multi-Biosignal Analysis for Epileptic Seizure Monitoring. *International Journal of Neural Systems, 27(1)*, 1650031.

Collard, S. S., & Ellis-Hill, C. (2017a). How do you exercise with epilepsy? Insights into the barriers and adaptations to successfully exercise with epilepsy. *Epilepsy and Behavior, Part A. 70*, 66-71.

Collard, S. S., & Ellis-Hill, C. (2017b). 'I'd rather you didn't come': The impact of stigma on exercising with epilepsy. *Journal of Health Psychology*, 1359105317729560.

Collard, S. S., & Marlow, C. (2016a). "it's such a vicious cycle": Narrative accounts of the sportsperson with epilepsy. *Psychology of Sport and Exercise, 24*, 56-64.

Collard, S. S., & Marlow, C. (2016b). The psychosocial impact of exercising with epilepsy: A narrative analysis. *Epilepsy & Behavior, 61*, 199-205.

Conradsen, I., Beniczky, S., Wolf, P., Jennum, P., & Sorensen, H. B. D. (2012). Evaluation of novel algorithm embedded in a wearable sEMG device for seizure detection. *Conference Proceedings: ... Annual International Conference of the IEEE Engineering in Medicine & Biology Society, 2012*, 2048-2051.

Cook, M. J., O'Brien, T. J., Berkovic, S. F., Murphy, M., Morokoff, A., Fabinyi, G., . . . Litewka, L. (2013). Prediction of seizure likelihood with a long-term, implanted seizure advisory system in patients with drug-resistant epilepsy: a first-in-man study. *The Lancet Neurology, 12*(6), 563-571.

Cuppens, K., Karsmakers, P., Van de Vel, A., Bonroy, B., Milosevic, M., Luca, S., . . . Van Huffel, S. (2014). Accelerometry-based home monitoring for detection of nocturnal hypermotor seizures based on novelty detection. *IEEE journal of biomedical and health informatics, 18*(3), 1026-1033.

de la Loge, C., Dimova, S., Mueller, K., Phillips, G., Durgin, T. L., Wicks, P., & Borghs, S. (2016). PatientsLikeMe Online Epilepsy Community: Patient characteristics and predictors of poor health-related quality of life. *Epilepsy & Behavior, 63*, 20-28.

Dennis, A. J. (2013). Identifying and responding to the reproductive decision-making needs of women with epilepsy. *Dissertation Abstracts International: Section B: The Sciences and Engineering, 74*(1-B(E)), No Pagination Specified.

DiIorio, C., Bamps, Y., Walker, E. R., & Escoffery, C. (2011). Results of a research study evaluating WebEase, an online epilepsy self-management program. *Epilepsy & Behavior, 22(3)*, 469-474.

DiIorio, C., Escoffery, C., McCarty, F., Yeager, K. A., Henry, T. R., Koganti, A., . . . Wexler, B. (2009a). Evaluation of WebEase: an epilepsy self-management Web site. *Health Education Research, 24(2)*, 185-197.

DiIorio, C., Escoffery, C., Yeager, K. A., McCarty, F., Henry, T. R., Koganti, A., . . . Price, P. (2009b). WebEase: development of a Web-based epilepsy self-management intervention. *Preventing Chronic Disease, 6(1)*, A28.

Dourado, A., Le Van Quyen, M., Schelter, B., Favaro, G., Schulze-Bonhage, A., Sales, S., & Navarro, V. (2009). Epilepsiae - Evolving platform for improving living expectation of patients suffering from ictal events. *Epilepsia, 50*, 210-211.

Dourado, A., Teixeira, C., Quyen, M. L. V., Schelter, B., Favaro, G., Schulz-Bonhage, A., . . . Ieee. (2014). Giving Hope to Refractory Epileptic Patients. *2014 Ist-Africa Conference Proceedings*.

Dumanis, S. B., French, J. A., Bernard, C., Worrell, G. A., & Fureman, B. E. (2017). Seizure Forecasting from Idea to Reality. Outcomes of the My Seizure Gauge Epilepsy Innovation Institute Workshop. *Eneuro, 4*(6), Nov-Dec.

Elger, C. E., & Mormann, F. (2013). Seizure prediction and documentation—two important problems. *The Lancet Neurology, 12*(6), 531-532. <https://doi.org/10.1016/s1474-4422(13)70092-9>

Ernst, L. d. L., Harden, C. L., Pennell, P. B., Llewellyn, N., Lau, C., Barnard, S., . . . French, J. A. (2016). Medication adherence in women with epilepsy who are planning pregnancy. *Epilepsia, 57(12)*, 2039-2044.

Escoffery, C., McGee, R., Bidwell, J., Sims, C., Thropp, E. K., Frazier, C., & Mynatt, E. D. (2018). A review of mobile apps for epilepsy self-management. *Epilepsy & Behavior, 81*, 62-69.

Fernandes, P. T., Snape, D. A., Beran, R. G., & Jacoby, A. (2011). Epilepsy stigma: What do we know and where next? *Epilepsy & Behavior, 22*(1), 55-62. <https://doi.org/10.1016/j.yebeh.2011.02.014>

Fisher, R. S. (2010). Tracking epilepsy with an electronic diary. *Acta Paediatrica, 99(4)*, 516-518.

Fisher, R. S. (2012). Therapeutic devices for epilepsy. *Annals of Neurology, 71(2)*, 157-168.

Fisher, R. S., Bartfeld, E., & Cramer, J. A. (2015). Use of an online epilepsy diary to characterize repetitive seizures. *Epilepsy & Behavior, 47*, 66-71.

Fisher, R. S., Blum, D. E., DiVentura, B., Vannest, J., Hixson, J. D., Moss, R., . . . French, J. A. (2012). Seizure diaries for clinical research and practice: limitations and future prospects. *Epilepsy & Behavior, 24(3)*, 304-310.

Fitzsimons, M., Doherty, C., Saris, J., Power, R., Lambert, V., & Varley, J. (2017). Co-designing Integrated Care Using Participatory Action Research [PAR]: The Epilepsy Partnership in Care [EPiC] Project. *International Journal of Integrated Care (IJIC), 17*, 1-2. <https://doi.org/10.5334/ijic.3643>

French, J., Harden, C., Pennell, P., Bagiella, E., Andreopoulos, E., Lau, C., . . . Davis, A. (2016). A prospective study of pregnancy in women with epilepsy seeking conception (the WEPOD study). *Neurology. Conference: 68th American Academy of Neurology Annual Meeting, AAN, 86*(16 SUPPL. 1).

Frings, L., Wagner, K., Maiwald, T., Carius, A., Schinkel, A., Lehmann, C., & Schulze-Bonhage, A. (2008). Early detection of behavioral side effects of antiepileptic treatment using handheld computers. *Epilepsy & Behavior, 13(2)*, 402-406.

Gauffin, H., Flensner, G., & Landtblom, A.-M. (2011). Living with epilepsy accompanied by cognitive difficulties: young adults' experiences. *Epilepsy & Behavior, 22(4)*, 750-758.

Ge, A., Gonzalez, E., Lee, S. W., Carmenate, Y., Collard, M., Dixon-Salazar, T., . . . Krauss, G. (2017). Seizure triggers in epilepsy patients: a national perspective. *Neurology. Conference: 69th American Academy of Neurology Annual Meeting, AAN, 88*(16 Supplement 1).

Geertsema, E. E., Thijs, R. D., Gutter, T., Vledder, B., Arends, J. B., Leijten, F. S., . . . Kalitzin, S. N. (2018). Automated video-based detection of nocturnal convulsive seizures in a residential care setting. *Epilepsia, 59 Suppl 1*, 53-60.

Gibney, E. (2015). The inside story on wearable electronics. *Nature, 528(7580)*, 26-28.

Gluckman, B. J., & Schevon, C. A. (2015). Seizure Prediction 6: [LINE SEPARATOR]From Mechanisms to Engineered Interventions for Epilepsy. *Journal of Clinical Neurophysiology, 32*(3), 181-187. <https://doi.org/10.1097/WNP.0000000000000184>

Goldenholz, D. M., Moss, R., Jost, D. A., Crone, N. E., Krauss, G., Picard, R., . . . Shafer, P. O. (2018). Common data elements for epilepsy mobile health systems. *Epilepsia, 59*(5), 1020-1026. <https://doi.org/doi:10.1111/epi.14066>

Gouravajhala, S. R., Wang, D., Khuon, L., & Bao, F. S. (2012). EpSMART: Epileptic Seizure Monitoring with Alerts in Real Time A Tablet-based Android Application for a Real-time Multi-modal Seizure Detection System. In J. Gao, W. Dubitzky, C. Wu, M. Liebman, R. Alhaij, L. Ungar, A. Christianson, & X. Hu (Eds.), *2012 Ieee International Conference on Bioinformatics and Biomedicine Workshops*.

Gubbi, J., Kusmakar, S., Rao, A. S., Yan, B., Obrien, T., & Palaniswami, M. (2016). Automatic Detection and Classification of Convulsive Psychogenic Nonepileptic Seizures Using a Wearable Device. *IEEE Journal of Biomedical & Health Informatics, 20(4)*, 1061-1072.

Hall, C. B., Lipton, R. B., Borkowski, T., & Fhaut, S. R. (2009a). Premonitory features predict seizures in an electronic diary study. *Epilepsia, 50*, 46-47.

Hall, C. B., Lipton, R. B., Tennen, H., & Haut, S. R. (2009b). Early follow-up data from seizure diaries can be used to predict subsequent seizures in same cohort by borrowing strength across participants. *Epilepsy & Behavior, 14(3)*, 472-475.

Harden, J., Tonberg, A., Chin, R. F., McLellan, A., & Duncan, S. (2015). 'If you're gonna die, you're gonna die': Young adults' perceptions of sudden unexpected death in epilepsy. *Chronic Illness, 11(3)*, 230-241.

Harper, K., Turchan, M., Riebau, D., Humphrey, A., Meyers, H., Zimmerman, E., . . . Charles, D. (2016). Physician satisfaction with teleneurology service provided via tablet technology. *Annals of Neurology, 80*, S186.

Haut, S. (2013). Predicting seizures: Are we there yet? *Epilepsy Currents, 13*(6), 276-278.

Haut, S. R., Hall, C. B., Borkowski, T., Tennen, H., & Lipton, R. B. (2012). Clinical features of the pre-ictal state: mood changes and premonitory symptoms. *Epilepsy & Behavior, 23(4)*, 415-421.

Haut, S. R., Hall, C. B., Borkowski, T., Tennen, H., & Lipton, R. B. (2013). Modeling seizure self-prediction: An e-diary study. *Epilepsia, 54*(11), 1960-1967.

Heldberg, B. E., Kautz, T., Leutheuser, H., Hopfengartner, R., Kasper, B. S., & Eskofier, B. M. (2015). Using wearable sensors for semiology-independent seizure detection - towards ambulatory monitoring of epilepsy. *Conference Proceedings: ... Annual International Conference of the IEEE Engineering in Medicine & Biology Society, 2015*, 5593-5596.

Helmy, A., Helmy, A., & Ieee. (2015). Seizario: Novel Mobile Algorithms for Seizure and Fall Detection. In *2015 Ieee Globecom Workshops*.

Herrmann, L. K., Welter, E., Berg, A. T., Perzynski, A. T., Van Doren, J. R., & Sajatovic, M. (2016). Epilepsy misconceptions and stigma reduction: Current status in Western countries. *Epilepsy & Behavior, 60*, 165-173. <https://doi.org/https://doi.org/10.1016/j.yebeh.2016.04.003>

Hixson, J. (2016). Use of a digital self-management platform for improving access in epilepsy patients. *Neurology. Conference: 68th American Academy of Neurology Annual Meeting, AAN, 86*(16 SUPPL. 1).

Hixson, J., Barnes, D., Parko, K., Durgin, T., Van Bebber, S., Wicks, P., & Graham, A. (2015a). The poem study: Patient usage and satisfaction with an online health management platform for epilepsy. *Epilepsy Currents, 15*, 210.

Hixson, J., Bertko, K., & Wicks, P. (2015b). User retention and utilization of an online digital health platform for epilepsy. *Neurology. Conference: 67th American Academy of Neurology Annual Meeting, AAN, 84*(no pagination).

Hixson, J. D., Barnes, D., Parko, K., Durgin, T., Van Bebber, S., Graham, A., & Wicks, P. (2015c). Patients optimizing epilepsy management via an online community: the POEM Study. *Neurology, 85(2)*, 129-136.

Horizon Scanning Research & Intelligence Centre. (2017). *Emerging technologies for the diagnosis, treatment and management of epilepsy*. Retrieved from

Jacoby, A. (1994). Felt versus enacted stigma: A concept revisited: Evidence from a study of people with epilepsy in remission. *Social Science & Medicine, 38*(2), 269-274. <https://doi.org/https://doi.org/10.1016/0277-9536(94)90396-4>

Jacoby, A., Ring, A., Whitehead, M., Marson, A., & Baker, G. A. (2014). Exploring loss and replacement of loss for understanding the impacts of epilepsy onset: a qualitative investigation. *Epilepsy & Behavior, 33*, 59-68.

Jacoby, A., Snape, D., & Baker, G. A. (2005). Epilepsy and social identity: the stigma of a chronic neurological disorder. *The Lancet Neurology, 4*(3), 171-178. <https://doi.org/https://doi.org/10.1016/S1474-4422(05)01014-8>

Johansson, D., Malmgren, K., & Alt Murphy, M. (2018). Wearable sensors for clinical applications in epilepsy, Parkinson's disease, and stroke: a mixed-methods systematic review. *Journal of Neurology, 265*(8), 1740-1752.

Jones, V. M., Veld, R. H. I., Tonis, T., Bults, R. B., van Beijnum, B., Widya, I., . . . Ieee. (2008). *Biosignal and Context Monitoring: Distributed Multimedia Applications of Body Area Networks in Healthcare*.

Jory, C., Shankar, R., Coker, D., McLean, B., Hanna, J., & Newman, C. (2016). Safe and sound? A systematic literature review of seizure detection methods for personal use. *Seizure, 36*, 4-15.

Kabir, A., Bedra, M., Cooper, L., Krumholz, A., & Finkelstein, J. (2015). Acceptance of a tele management system to support epilepsy self-care. *Neurology. Conference: 67th American Academy of Neurology Annual Meeting, AAN, 84*(no pagination).

Kerr, C., Nixon, A., & Angalakuditi, M. (2011). The impact of epilepsy on children and adult patients' lives: development of a conceptual model from qualitative literature. *Seizure, 20(10)*, 764-774.

Kilinc, S., & Campbell, C. (2009). "It shouldn't be something that's evil, it should be talked about": a phenomenological approach to epilepsy and stigma. *Seizure, 18(10)*, 665-671.

Kilinc, S., Campbell, C., Guy, A., & van Wersch, A. (2018). Epilepsy, identity, and the experience of the body. *Epilepsy & Behavior, 89*, 42-47.

Kilinc, S., van Wersch, A., Campbell, C., & Guy, A. (2017). The experience of living with adult-onset epilepsy. *Epilepsy & Behavior, 73*, 189-196.

Kingwell, K. (2013). Epilepsy: Implantable device advises patients with epilepsy of seizure likelihood. *Nature Reviews Neurology, 9*(6), 297.

Kramer, U., Kipervasser, S., Shlitner, A., & Kuzniecky, R. (2011). A novel portable seizure detection alarm system: preliminary results. *Journal of Clinical Neurophysiology, 28(1)*, 36-38.

Kurada, A. V., Srinivasan, T., Hammond, S., Ulate-Campos, A., & Bidwell, J. (2019). Seizure detection devices for use in antiseizure medication clinical trials: A systematic review. *Seizure, 66*, 61-69.

Kusmakar, S., Karmakar, C., Yan, B., Obrien, T., Muthuganapathy, R., & Palaniswami, M. (2018). Automated Detection of Convulsive Seizures Using a Single Wrist-Worn Accelerometer Device. *IEEE Transactions on Biomedical Engineering, 11*, 11.

Lapalme-Remis, S., Kumbamu, A., Young, C., Aase, L., Jette, N., & Leep Hunderford, A. (2017). Experiences with epilepsy treatments: A qualitative content analysis of online patient support group discussions. *Canadian Journal of Neurological Sciences, 44 (Supplement 2)*, S22.

Lawther, L., Dolk, H., Sinclair, M., & Morrow, J. (2018). The preconception care experiences of women with epilepsy on sodium valproate. *Seizure, 59*, 82-89.

Le, S., Shafer, P. O., Bartfeld, E., & Fisher, R. S. (2011). An online diary for tracking epilepsy. *Epilepsy & Behavior, 22(4)*, 705-709.

Leenen, L. A. M., Wijnen, B. F. M., de Kinderen, R. J. A., van Heugten, C. M., Evers, S. M. A. A., & Majoie, M. H. J. M. (2016). Are people with epilepsy using eHealth-tools? *Epilepsy & Behavior, 64(Pt A)*, 268-272.

Leenen, L. A. M., Wijnen, B. F. M., van Haastregt, J. C. M., de Kinderen, R. J. A., Evers, S. M. A. A., Majoie, M. H. J. M., & van Heugten, C. M. (2017). Process evaluation of a multi-component self-management intervention for adults with epilepsy (ZMILE study). *Epilepsy & Behavior, 73*, 64-70.

Lehnertz, K. (2001). Seizure anticipation techniques: state of the art and future requirements. In *Proceedings of the 23rd Annual International Conference of the Ieee Engineering in Medicine and Biology Society, Vols 1-4: Building New Bridges at the Frontiers of Engineering and Medicine* (Vol. 23, pp. 4121-4123).

Leppik, I. E. (2006). Epilepsy in the Elderly. *Epilepsia, 47*, 65-70. <https://doi.org/10.1111/j.1528-1167.2006.00664.x>

Litt, B. (2001). Engineering devices to treat epilepsy: A clinical perspective. In *Proceedings of the 23rd Annual International Conference of the Ieee Engineering in Medicine and Biology Society, Vols 1-4: Building New Bridges at the Frontiers of Engineering and Medicine* (Vol. 23, pp. 4124-4128).

Litt, B., & Echauz, J. (2002). Prediction of epileptic seizures. *Lancet Neurology, 1*(1), 22-30.

Liu, X., Wang, R., Zhou, D., & Hong, Z. (2016a). Feasibility and acceptability of smartphone applications for seizure self-management in China: Questionnaire study among people with epilepsy. *Epilepsy & Behavior, 55*, 57-61.

Liu, X., Wang, R., Zhou, D., & Hong, Z. (2016b). Smartphone applications for seizure care and management in children and adolescents with epilepsy: Feasibility and acceptability assessment among caregivers in China. *Epilepsy Research, 127*, 1-5.

Llewellyn, N., Harden, C. L., French, J., Pennell, P. B., Bartfeld, E., Davis, A. R., . . . Bagiella, E. (2013). Maintenance of subject adherence to daily diary entry facilitated by use of a mobile application in the wepod study. *Epilepsy Currents, 13*, 69-70.

Lockman, J., Fisher, R. S., & Olson, D. M. (2011). Detection of seizure-like movements using a wrist accelerometer. *Epilepsy & Behavior, 20(4)*, 638-641.

Lua, P. L., & Neni, W. S. (2013). Health-related quality of life improvement via telemedicine for epilepsy: printed versus SMS-based education intervention. *Quality of Life Research, 22*(8), 2123-2132. <https://doi.org/10.1007/s11136-013-0352-6>

Lyons, B., & Dolezal, L. (2017). Shame, stigma and medicine. *Medical humanities, 43*(4), 208-210.

Mahrer-Imhof, R., Jaggi, S., Bonomo, A., Hediger, H., Eggenschwiler, P., Kramer, G., & Oberholzer, E. (2013). Quality of life in adult patients with epilepsy and their family members. *Seizure-European Journal of Epilepsy, 22*(2), 128-135. <https://doi.org/10.1016/j.seizure.2012.11.012>

Maiwald, T., Blumberg, J., Timmer, J., & Schulze-Bonhage, A. (2011). Are prodromes preictal events? A prospective PDA-based study. *Epilepsy & Behavior, 21(2)*, 184-188.

Martin, R., Vogtle, L., Gilliam, F., & Faught, E. (2003). Health-related quality of life in senior adults with epilepsy: what we know from randomized clinical trials and suggestions for future research. *Epilepsy & Behavior, 4*(6), 626-634.

Martin, R., Vogtle, L., Gilliam, F., & Faught, E. (2005). What are the concerns of older adults living with epilepsy? *Epilepsy & Behavior, 7*(2), 297-300. <https://doi.org/https://doi.org/10.1016/j.yebeh.2005.05.003>

McLaughlin, D. P., Pachana, N. A., & McFarland, K. (2008). Stigma, seizure frequency and quality of life: The impact of epilepsy in late adulthood. *Seizure, 17*(3), 281-287. <https://doi.org/https://doi.org/10.1016/j.seizure.2007.09.001>

McLean, B., Shankar, R., Hanna, J., Jory, C., & Newman, C. (2017). Sudden unexpected death in epilepsy: measures to reduce risk. *Practical Neurology, 17*(1), 13-20.

Mengoni, S. E., Gates, B., Parkes, G., Wellsted, D., Barton, G., Ring, H., . . . Durand, M.-A. (2016). "Sometimes, it just stops me from doing anything": A qualitative exploration of epilepsy management in people with intellectual disabilities and their carers. *Epilepsy & Behavior, 64(Pt A)*, 133-139.

Meritam, P., Ryvlin, P., & Beniczky, S. (2018). User-based evaluation of applicability and usability of a wearable accelerometer device for detecting bilateral tonic-clonic seizures: A field study. *Epilepsia, 59 Suppl 1*, 48-52.

Miller, W. R., Bakas, T., & Buelow, J. M. (2014). Problems, needs, and useful strategies in older adults self-managing epilepsy: Implications for patient education and future intervention programs. *Epilepsy and Behavior, 31*, 25-30.

Moccia, M., Brigo, F., Tedeschi, G., Bonavita, S., & Lavorgna, L. (2018). Neurology and the Internet: a review. *Neurological Sciences, 39*(6), 981-987. <https://doi.org/10.1007/s10072-018-3339-9>

Monzoni, C., & Reuber, M. (2009). Conversational displays of coping resources in clinical encounters between patients with epilepsy and neurologists: A pilot study. *Epilepsy & Behavior, 16*(4), 652-659. <https://doi.org/10.1016/j.yebeh.2009.08.004>

Mormann, F., Andrzejak, R. G., Elger, C. E., & Lehnertz, K. (2006). Seizure prediction: the long and winding road. *Brain, 130*(2), 314-333.

Morrell, M. J. (2009). Results of a multicenter double blinded randomized controlled pivotal investigation of the RNSz System for treatment of intractable partial epilepsy in adults. *Epilepsia, 50*, 490.

Myers, M. H., Threatt, M., Solies, K. M., McFerrin, B. M., Hopf, L. B., Birdwell, J. D., & Sillay, K. A. (2016). Ambulatory seizure monitoring: From concept to prototype device. *Annals of Neurosciences, 23*(2), 100-111.

Nagaraj, V., Lee, S., Krook-Magnuson, E., Soltesz, I., Benquet, P., Irazoqui, P., & Netoff, T. (2015). The future of seizure prediction and intervention: closing the loop. *Journal of clinical neurophysiology: official publication of the American Electroencephalographic Society, 32*(3), 194.

Newman, C., Shankar, R., Hanna, J., McLean, B., Osland, A., Milligan, C., . . . Walker, M. (2016). Developing an Evidence-Based Epilepsy Risk Assessment eHealth Solution: From Concept to Market. *JMIR Research Protocols, 5(2)*, e82.

Nijsen, T. M., Arends, J. B., Griep, P. A., & Cluitmans, P. J. (2005). The potential value of three-dimensional accelerometry for detection of motor seizures in severe epilepsy. *Epilepsy & Behavior, 7*(1), 74-84.

Ninnoni, J. P. K. (2019). A qualitative study of the communication and information needs of people with learning disabilities and epilepsy with physicians, nurses and carers. *BMC Neurology, 19*(1), 12.

Olsen, L., Rasmussen, P., & Stubbings, V. (2017). "Seizure monitoring onmyapp" a nursing approach in the implementation of a seizure app. perspectives from clinical practice. *Epilepsia, 58 (Supplement 5)*, S11.

Ozanne, A., Johansson, D., Hallgren Graneheim, U., Malmgren, K., Bergquist, F., & Alt Murphy, M. (2017). Wearables in epilepsy and Parkinson's disease-A focus group study. *Acta Neurologica Scandinavica.*

Page, R., Knight, M., & Symon, K. (2016). Holistic application of new technology to the management of epilepsy: The epilepsy networks project. *Epilepsia, 57*, 43.

Page, R., Knight, M., & Symon, K. (2018a). 1 year pilot study of a technology-enabled platform for supporting patients with epilepsy. *Epilepsia, 59 (Supplement 3)*, S342.

Page, R., Knight, M., & Symon, K. (2018b). Epilepsy technotherapy - Results of a 1 year pilot study. *Neurology. Conference: 70th Annual Meeting of the American Academy of Neurology, AAN, 90*(15 Supplement 1).

Page, R., Shankar, R., McLean, B. N., Hanna, J., & Newman, C. (2018c). Digital care in epilepsy: a conceptual framework for technological therapies. *Frontiers in neurology, 9*, 99.

Pandher, P. S., & Bhullar, K. K. (2016). Smartphone applications for seizure management. *Health Informatics Journal, 22(2)*, 209-220.

Patel, A. D., Moss, R., Rust, S. W., Patterson, J., Strouse, R., Gedela, S., . . . Lin, S. M. (2016). Patient-centered design criteria for wearable seizure detection devices. *Epilepsy & Behavior, 64(Pt A)*, 116-121.

Patel, S., Mancinellil, C., Dalton, A., Patritti, B., Pang, T., Schachter, S., . . . Ieee. (2009). *Detecting Epileptic Seizures Using Wearable Sensors*.

Patterson, A. L., Mudigoudar, B., Fulton, S., McGregor, A., Poppel, K. V., Wheless, M. C., . . . Wheless, J. W. (2015). SmartWatch by SmartMonitor: Assessment of Seizure Detection Efficacy for Various Seizure Types in Children, a Large Prospective Single-Center Study. *Pediatric Neurology, 53(4)*, 309-311.

Patterson, V., & Bingham, E. (2005). Telemedicine for Epilepsy: A Useful Contribution. *Epilepsia, 46*(5), 614-615.

Pembroke, S., Higgins, A., Pender, N., & Elliott, N. (2017). Becoming comfortable with "my" epilepsy: Strategies that patients use in the journey from diagnosis to acceptance and disclosure. *Epilepsy & Behavior, 70(Pt A)*, 217-223.

Pennell, P., French, J., Harden, C. L., Bartfeld, E., Davis, A. R., Llewellyn, N. G., . . . Keenan, H. A. (2012). Evaluation of a mobile application tool in the wepod study. *Epilepsy Currents. Conference: 65th Annual Meeting of the American Epilepsy Society, AES. Baltimore, MD United States. Conference Publication:, 12*(1 SUPPL. 1).

Perzynski, A. T., Ramsey, R. K., Colon-Zimmermann, K., Cage, J., Welter, E., & Sajatovic, M. (2017). Barriers and facilitators to epilepsy self-management for patients with physical and psychological co-morbidity. *Chronic Illness, 13(3)*, 188-203.

Picard, R. W., Migliorini, M., Caborni, C., Onorati, F., Regalia, G., Friedman, D., & Devinsky, O. (2017). Wrist sensor reveals sympathetic hyperactivity and hypoventilation before probable SUDEP. *Neurology, 89*(6), 633-635.

Poh, M. Z., Loddenkemper, T., Reinsberger, C., Swenson, N. C., Goyal, S., Sabtala, M. C., . . . Picard, R. W. (2012). Convulsive seizure detection using a wrist‐worn electrodermal activity and accelerometry biosensor. *Epilepsia, 53*(5), e93-e97.

Polak, E., Apfel, A., Privitera, M., Buse, D., & Haut, S. (2014). Daily diaries in epilepsy research: Does electronic format improve adherence? *Epilepsy Currents, 14*, 180.

Popescu, M., Supeanu, A., Grigorean, V., Strambu, V., Popescu, G., & Plesea, E. (2013). Portable device for real time monitoring and warning of epileptic seizures. *Epilepsy and Behavior, 28 (2)*, 332.

Poppel, K. V., Fulton, S. P., McGregor, A., Ellis, M., Patters, A., & Wheless, J. (2013). Prospective study of the Emfit movement monitor. *Journal of child neurology, 28*(11), 1434-1436.

Power, K., Fitzsimons, M., White, M., Cavalleri, G., Dunleavey, B., Corbridge, R., . . . McCormack, M. (2017). A Patient Portal Providing Individualised Services and Care in Epilepsy (PISCES). *International Journal of Integrated Care (IJIC), 17*, 1-2. <https://doi.org/10.5334/ijic.3373>

Privitera, M., Polak, E., Fleck, A., & Haut, S. (2015). Stress management intervention for living with epilepsy: Results of a RCT. *Epilepsia, 56*, 24.

Raam, G., & Sasikala, R. (2016). Detection and prediction of seizures using a wrist-based wearable platform. *International Journal of Computer Technology Applications, 9*(37), 193-203.

Ranganathan, L. N., Aadhimoolam Chinnadurai, S., Samivel, B., Kesavamurthy, B., & Mehndiratta, M. M. (2015). Application of mobile phones in epilepsy care. *International Journal of Epilepsy, 2*(1), 28-37.

Raty, L. K., & Wilde-Larsson, B. M. (2011). Patients' perceptions of living with epilepsy: a phenomenographic study. *Journal of Clinical Nursing, 20(13-14)*, 1993-2002.

Räty, L. K. A., Larsson, G., Starrin, B., & Larsson, B. M. W. (2009). Epilepsy patients' conceptions of epilepsy as a phenomenon. *Journal of Neuroscience Nursing, 41*(4), 201-210.

Raty, L. K. A., Soderfeldt, B. A., & Larsson, B. M. W. (2007). Daily life in epilepsy: Patients' experiences described by emotions. *Epilepsy & Behavior, 10*(3), 389-396. <https://doi.org/10.1016/j.yebeh.2007.02.003>

Rawlings, G. H., Brown, I., & Reuber, M. (2018a). Narrative analysis of written accounts about living with epileptic or psychogenic nonepileptic seizures. *Seizure, 62*, 59-65.

Rawlings, G. H., Brown, I., Stone, B., & Reuber, M. (2017). Written accounts of living with epilepsy: A thematic analysis. *Epilepsy & Behavior, 72*, 63-70.

Rawlings, G. H., Brown, I., Stone, B., & Reuber, M. (2018b). Written Accounts of Living With Epilepsy or Psychogenic Nonepileptic Seizures: A Thematic Comparison. *Qualitative Health Research, 28*(6), 950-962. <https://doi.org/10.1177/1049732317748897>

Reeder, B., & David, A. (2016). Health at hand: A systematic review of smart watch uses for health and wellness. *Journal of Biomedical Informatics, 63*, 269-276.

Regalia, G., Onorati, F., Migliorini, M., & Picard, R. (2015). *An improved wrist-worn convulsive seizure detector based on accelerometry and electrodermal activity sensors.* Paper presented at the American Epilepsy Society annual meeting 2015.

Reis, R., & Meinardi, H. (2002). ILAE/WHO “Out of the Shadows Campaign” Stigma: does the flag identify the cargo? *Epilepsy & Behavior, 3*(6, Supplement 2), 33-37. <https://doi.org/https://doi.org/10.1016/S1525-5050(02)00546-2>

Rhodes, P. J., Small, N. A., Ismail, H., & Wright, J. P. (2008). 'What really annoys me is people take it like it's a disability', epilepsy, disability and identity among people of Pakistani origin living in the UK. *Ethnicity & Health, 13*(1), 1-21.

Ring, A., Jacoby, A., Baker, G. A., Marson, A., & Whitehead, M. M. (2016). Does the concept of resilience contribute to understanding good quality of life in the context of epilepsy? *Epilepsy and Behavior, 56*, 153-164.

Roach, R. (2017). Epilepsy, digital technology and the black-boxed self. *New Media & Society*, 1461444817736926.

Rodriguez-Villegas, E., Aguilar-Pelaez, E., Chen, G., & Duncan, J. (2009). Apnea detector to prevent SUDEP. *Epilepsia, 50*, 37-38.

Rossi, M. A., Monica, N., Babiarz, K., Cendejas, L., Hanson, R., Tshionyi, M., . . . Jain, M. (2015). A mobile health-intensive comprehensive care delivery model for amplifying outreach for refractory epilepsy and co-morbid mood disorders. *Epilepsy Currents, 15*, 222-223.

Ryan, S., & Raisanen, U. (2012). "The brain is such a delicate thing": an exploration of fear and seizures among young people with epilepsy. *Chronic Illness, 8(3)*, 214-224.

Sajatovic, M., Jobst, B. C., Shegog, R., Bamps, Y. A., Begley, C. E., Fraser, R. T., . . . Kobau, R. (2017). The Managing Epilepsy Well Network:: Advancing Epilepsy Self-Management. *American Journal of Preventive Medicine, 52(3S3)*, S241-S245.

Salem, O., Rebhi, Y., Boumaza, A., Mehaoua, A., & Ieee. (2014). Detection of Nocturnal Epileptic Seizures Using Wireless 3-D Accelerometer Sensors. *2014 Ieee 16th International Conference on E-Health Networking, Applications and Services (Healthcom)*, 237-242.

Sallay, V., Martos, T., Chatfield, S. L., & Dull, A. (2019). Strategies of Dyadic Coping and Self-Regulation in the Family Homes of Chronically III Persons: A Qualitative Research Study Using the Emotional Map of the Home Interview Method. *Frontiers in Psychology, 10*. <https://doi.org/10.3389/fpsyg.2019.00403>

Sareen, S., Sood, S. K., & Gupta, S. K. (2016). An Automatic Prediction of Epileptic Seizures Using Cloud Computing and Wireless Sensor Networks. *Journal of Medical Systems, 40 (11) (no pagination)*(226).

Scambler, G. (1989). Epilepsy. Tavistock. In: London.

Scambler, G. (2004). Re-framing Stigma: Felt and Enacted Stigma and Challenges to the Sociology of Chronic and Disabling Conditions. *Social Theory & Health, 2*(1), 29-46. <https://doi.org/10.1057/palgrave.sth.8700012>

Schwartz, T. H. (2007). Neurovascular coupling and epilepsy: hemodynamic markers for localizing and predicting seizure onset. *Epilepsy Currents, 7*(4), 91-94.

Scott, A. J., Sharpe, L., Thayer, Z., Miller, L. A., Wong, T., Parratt, K., & Nikpour, A. (2018). A qualitative examination and theoretical model of anxiety in adults with epilepsy. *Epilepsy and Behavior, 85*, 95-104.

Serhani, M. A., Menshawy, M. E., & Benharref, A. (2016). SME2EM: Smart mobile end-to-end monitoring architecture for life-long diseases. *Computers in Biology & Medicine, 68*, 137-154.

Shankar, R., Newman, C., McLean, B., Anderson, T., & Obe, J. H. (2015). Can technology help reduce risk of harm in patients with epilepsy? *British Journal of General Practice, 65*(638), 448-449.

Shegog, R., Bamps, Y. A., Patel, A., Kakacek, J., Escoffery, C., Johnson, E. K., & Ilozumba, U. O. (2013). Managing Epilepsy Well: Emerging e-Tools for epilepsy self-management. *Epilepsy & Behavior, 29(1)*, 133-140.

Shegog, R., & Begley, C. (2015). Development and evaluation of a clinic based decision support system for epilepsy self-management in the USA. *Epilepsia, 56*, 187-188.

Shi, Y., Wang, S. Q., Ying, J., Zhang, M. L., Liu, P. C., Zhang, H. H., & Sun, J. (2017). Correlates of perceived stigma for people living with epilepsy: A meta-analysis. *Epilepsy & Behavior, 70*, 198-203. <https://doi.org/10.1016/j.yebeh.2017.02.022>

Shostak, S., & Fox, N. S. (2012). Forgetting and remembering epilepsy: collective memory and the experience of illness. *Sociology of health & illness, 34*(3), 362-378.

Sleeth, C., Drake, K., Labiner, D. M., & Chong, J. (2016). Felt and enacted stigma in elderly persons with epilepsy: A qualitative approach. *Epilepsy & Behavior, 55*, 108-112.

Stacey, W. C., & Litt, B. (2008). Technology insight: neuroengineering and epilepsy—designing devices for seizure control. *Nature clinical practice Neurology, 4*(4), 190-201.

Stepney, M., Kirkpatrick, S., Locock, L., Prinjha, S., & Ryan, S. (2018). A licence to drive? Neurological illness, loss and disruption. *Sociology of health & illness, 40*(7), 1186-1199. <https://doi.org/doi:10.1111/1467-9566.12754>

Stigwall, J., Hildeman, A., Wipenmyr, J., Pettersson, T., Malmgren, K., & Rydenhag, B. (2011). Detection of epileptic seizures by pattern recognition from wireless accelerometer data. *Epilepsia, 52*, 40.

Sun, F. T., & Morrell, M. J. (2014). Closed-loop neurostimulation: the clinical experience. *Neurotherapeutics, 11(3)*, 553-563.

Tebartz van Elst, L., Baker, G., & Kerr, M. (2009). The psychosocial impact of epilepsy in older people. *Epilepsy & Behavior, 15*(2, Supplement 1), S17-S19. <https://doi.org/https://doi.org/10.1016/j.yebeh.2009.03.015>

Thanaviratananich, S., Van Ness, P., Haneef, Z., & Chen, D. (2017). The predictability of smartphone video in distinguishing epileptic and nonepileptic seizures. *Neurology. Conference: 69th American Academy of Neurology Annual Meeting, AAN, 88*(16 Supplement 1).

Thompson, D., Thomas, H., Solomon, J., Nashef, L., & Kendall, S. (2008). Chronic illness, reproductive health and moral work: women's experiences of epilepsy. *Chronic Illness, 4(1)*, 54-64.

Thompson, R., Linehan, C., Glynn, M., & Kerr, M. P. (2013). A qualitative study of carers' and professionals' views on the management of people with intellectual disability and epilepsy: a neglected population. *Epilepsy & Behavior, 28(3)*, 379-385.

Todd, L., Burford, J., & Ireland, C. (2017). Curated epilepsy content to foster selfmanagement: Myepilepsykey. *Epilepsia, 58 (Supplement 5)*, S137-S138.

Unger, W. R., & Buelow, J. M. (2009). Hybrid concept analysis of self-management in adults newly diagnosed with epilepsy. *Epilepsy & Behavior, 14(1)*, 89-95.

Vadrot, A. B. M. (2013). "Rather a manager and networker than a researcher'': converging technologies in the clinic. *Innovation-the European Journal of Social Science Research, 26*(4), 376-397. <https://doi.org/10.1080/13511610.2013.805296>

van Andel, J., Ungureanu, C., Arends, J., Tan, F., Van Dijk, J., Petkov, G., . . . Leijten, F. (2017). Multimodal, automated detection of nocturnal motor seizures at home: Is a reliable seizure detector feasible? *Epilepsia open, 2*(4), 424-431. <https://doi.org/10.1002/epi4.12076>

Van Bussel, M. J., Penders, J., & Arends, J. (2012). Real-time detection of major epileptic seizures: Feasibility study of a miniaturized wireless cardiac monitor. *Epilepsy Currents. Conference: 65th Annual Meeting of the American Epilepsy Society, AES. Baltimore, MD United States. Conference Publication:, 12*(1 SUPPL. 1).

Van de Vel, A., Cuppens, K., Bonroy, B., Milosevic, M., Jansen, K., Van Huffel, S., . . . Ceulemans, B. (2016a). Non-EEG seizure detection systems and potential SUDEP prevention: state of the art: review and update. *Seizure, 41*, 141-153.

Van de Vel, A., Cuppens, K., Bonroy, B., Milosevic, M., Jansen, K., Van Huffel, S., . . . Ceulemans, B. (2013). Non-EEG seizure-detection systems and potential SUDEP prevention: state of the art. *Seizure, 22*(5), 345-355.

Van de Vel, A., Milosevic, M., Bonroy, B., Cuppens, K., Lagae, L., Vanrumste, B., . . . Ceulemans, B. (2016b). Long-term accelerometry-triggered video monitoring and detection of tonic-clonic and clonic seizures in a home environment: Pilot study. *Epilepsy & Behavior Case Reports, 5*, 66-71.

Varley, J., Power, R., Saris, J., & Fitzsimons, M. (2017). Co-designing patient-centred care using participatory action research [par] - The epilepsy partnership in care [epic] project. *International Journal for Quality in Health Care, 29 (Supplement 1)*, 8-9.

Velez, M., Fisher, R. S., Bartlett, V., & Le, S. (2016). Tracking generalized tonic-clonic seizures with a wrist accelerometer linked to an online database. *Seizure, 39*, 13-18.

Walker, E. R., Bamps, Y., Burdett, A., Rothkopf, J., & Diiorio, C. (2012). Social support for self-management behaviors among people with epilepsy: a content analysis of the WebEase program. *Epilepsy & Behavior, 23(3)*, 285-290.

Walker, E. R., Barmon, C., McGee, R. E., Engelhard, G., Sterk, C. E., DiIorio, C., & Thompson, N. J. (2014a). Perspectives of adults with epilepsy and their support persons on self-management support. *Qualitative Health Research, 24(11)*, 1553-1566.

Walker, E. R., Barmon, C., McGee, R. E., Engelhard, G., Sterk, C. E., DiIorio, C., & Thompson, N. J. (2015). A dyadic model of living with epilepsy based on the perspectives of adults with epilepsy and their support persons. *Epilepsy & Behavior, 53*, 1-9.

Walker, E. R., Engelhard, G., Barmon, C., McGee, R. E., Sterk, C. E., DiIorio, C., & Thompson, N. J. (2014b). A mixed methods analysis of support for self-management behaviors: Perspectives of people with epilepsy and their support providers. *Epilepsy & Behavior, 31*, 152-159. <https://doi.org/https://doi.org/10.1016/j.yebeh.2013.11.023>

Walker, E. R., Wexler, B., Dilorio, C., Escoffery, C., McCarty, F., & Yeager, K. A. (2009). Content and characteristics of goals created during a self-management intervention for people with epilepsy. *Journal of Neuroscience Nursing, 41(6)*, 312-321.

Weckesser, A., & Denny, E. (2013). Women living with epilepsy, experiences of pregnancy and reproductive health: a review of the literature. *Seizure, 22(2)*, 91-98.

Weckesser, A., Denny, E., & Network, E. M. C. (2017). Re-working biographies: Women's narratives of pregnancy whilst living with epilepsy. *Social Science & Medicine, 185*, 110-117.

Wedlund, E. W., Nilsson, L., Tomson, T., & Erdner, A. (2013). What is important in rehabilitation for persons with epilepsy? Experiences from focus group interviews with patients and staff. *Epilepsy & Behavior, 28(3)*, 347-353.

Widnes, S. F., Schjott, J., & Granas, A. G. (2012). Risk perception and medicines information needs in pregnant women with epilepsy--a qualitative study. *Seizure, 21(8)*, 597-602.

Yasam, V. R., Jakki, S. L., Senthil, V., Jawahar, N., Vengal Rao, P., & Chalichem, N. S. S. (2018). An overview of non-drug therapies for the treatment of epilepsy. *Indian Journal of Pharmaceutical Sciences, 80*(2), 223-234.

Yennadiou, H., & Wolverson, E. (2017). The experience of epilepsy in later life: A qualitative exploration of illness representations. *Epilepsy & Behavior, 70(Pt A)*, 87-93.

Yuen, A. W. C., Keezer, M. R., & Sander, J. W. (2018). Epilepsy is a neurological and a systemic disorder. *Epilepsy & Behavior, 78*, 57-61. <https://doi.org/https://doi.org/10.1016/j.yebeh.2017.10.010>

Zhang, S., Chen, R., Javaherian, K., Groenendyk, J., Pan, M., & Maccotta, L. (2017). Catching and counting seizures for patients with epilepsy using an automated telemedicine platform - EpxEpilepsy. *Neurology. Conference: 69th American Academy of Neurology Annual Meeting, AAN, 88*(16 Supplement 1).

Zhao, X., & Lhatoo, S. D. (2018). Seizure detection: do current devices work? And when can they be useful? *Current Neurology & Neuroscience Reports, 18*(7), 40.
